# The chromatin accessibility landscape during early maize seed development

**DOI:** 10.1111/tpj.70073

**Published:** 2025-03-24

**Authors:** Guang Ming Zheng, Jia Wen Wu, Jun Li, Ya Jie Zhao, Chao Zhou, Ru Chang Ren, Yi Ming Wei, Xian Sheng Zhang, Xiang Yu Zhao

**Affiliations:** ^1^ State Key Laboratory of Crop Biology, College of Life Sciences Shandong Agricultural University Taian Shandong 271018 China

**Keywords:** accessible chromatin region (ACR), stage‐specific ACRs, transcription factor footprints, transcription regulatory network, maize seed development, *Zea mays*

## Abstract

*Cis*‐regulatory elements (CREs) are enriched in *accessible chromatin regions* (ACRs) of eukaryotes. Despite extensive research on genome‐wide ACRs in various plant tissues, the global impact of these changes on developmental processes in maize seeds remains poorly understood. In this study, we employed the assay for transposase‐accessible chromatin sequencing (ATAC‐seq) to reveal the chromatin accessibility profile throughout the genome during the early stages of maize seed development. We identified a total of 37 952 to 59 887 high‐quality ACRs in maize seeds at 0 to 8 days after pollination (DAP). Furthermore, we examined the correlation between the identified ACRs and gene expression. We observed a positive correlation between the open degree of promoter‐ACRs and the expression of most genes. Moreover, we identified binding footprints of numerous transcription factors (TFs) within chromatin accessibility regions and revealed key TF families involved in different stages. Through the footprints of accessible chromatin regions, we predicted transcription factor regulatory networks during early maize embryo development. Additionally, we discovered that DNA sequence diversity was notably reduced at ACRs, yet trait‐associated SNPs were more likely to be located within ACRs. We edited the ACR containing the trait‐associated SNP of *NKD1*. Both *NKD1* ^*pro*^ *‐1* and *NKD1* ^*pro*^ *‐2* showed phenotypes corresponding to the trait‐associated SNP. Our results suggest that alterations in chromatin accessibility play a crucial role in maize seed development and highlight the potential contribution of open chromatin regions to advancements in maize breeding.

## Significance Statement

Despite extensive research on genome‐wide accessible chromatin regions (ACRs) in various plant tissues, the global impact of these changes on developmental processes in maize seeds remains poorly understood. In this study, we employed the assay for transposase‐accessible chromatin sequencing (ATAC‐seq) to uncover the chromatin accessibility profile throughout the genome during the early stages of maize seed development and to investigate the ACR changes regulating seed development.

## INTRODUCTION

Open chromatin is closely related to gene expression and regulatory sequences are predominantly located in *accessible chromatin regions* (ACRs) (Shu et al., 2012; Shu et al., 2013; Thurman et al., 2012). Many *cis‐regulatory elements* (CREs) are located within ACRs and regulate plant development through spatiotemporal changes in accessibility (Marand et al., 2021). ACRs are highly enriched in the promoter regions, and their accessibility is largely positively correlated with the expression level of associated genes (Rodgers‐Melnick et al., 2016). In addition, many ACRs located in intergenic regions can regulate the expression of target genes through long‐range chromatin interactions (Ouyang et al., 2020; Ricci et al., 2019; Sun et al., 2020). An active intergenic ACR, the Hopscotch retrotransposon, is inserting 60 kilobase pair (kb) upstream of *teosinte branched1* (*tb1*), which enhances *tb1* transcription and converts teosinte to maize architecture (Doebley et al., 1997; Studer et al., 2011). Transcription factors (TFs) can bind to ACRs that contain motifs to regulate gene expression. The sequence diversity of ACRs results in different binding abilities of TFs, which is an important factor contributing to population diversity (Galli et al., 2018; Tu et al., 2020). Uncovering the regulatory mechanisms of complex traits is essential for crop improvement, and genome‐wide identification of open chromatin regions offers new insights into these mechanisms (Zhu et al., 2024). *Trans*‐acting factor works in tandem with ACRs and epigenetic modifications to regulate gene expression, with a strong correlation observed between chromatin accessibility and gene expression (Liu et al., 2023; Wang et al., 2022). Furthermore, editing these ACRs can alter traits by changing gene expression (Hendelman et al., 2021; Wang et al., 2021).

Maize seed plays a crucial role as a source of food and fuel. Its intricate and hierarchical structure, encompassing various cell types, offers an excellent model for investigating seed development in angiosperms. The maize seed's endosperm, being the most vital storage tissue, undergoes three main cytological stages within 8 days of double fertilization: coenocyte (1 to 3 DAP), cellularization (3 to 4 DAP), and differentiation (early: 4 to 6 DAP, late: 8 to 12 DAP) (Leroux et al., 2014; Yi et al., 2019). The opening of chromatin differs among various cell types, influencing cellular fate (Marand et al., 2021). The assay for transposase‐accessible chromatin sequencing (ATAC‐seq) can efficiently and quickly detect chromatin landscapes from samples of different sizes. Previous studies have most centered on transcriptome analysis during seed development, while limited attention has been paid to the investigation of chromatin accessibility (Chen et al., 2014; Yi et al., 2019). Here, we employed ATAC‐Seq to draw the chromatin accessibility profile during the early stages of maize seed development. We identified a series of ACRs during maize seed development and analyzed transcription factor footprints in ACRs. For various stages of development, both the quantity and extent of ACRs were significantly disparate. The binding of transcription factors to ACRs was found to be related to the developmental stage and was characterized by differences in both the quantity and strength. The sequences of ACRs were found to be conserved within a population, but mutations in these sequences could result in phenotypic diversification. As a result, these findings can be utilized to establish novel molecular markers for genetic variation, thereby enabling advancements in molecular breeding.

## RESULTS

### Characterization of chromatin accessibility in maize seed

**Figure 1:**
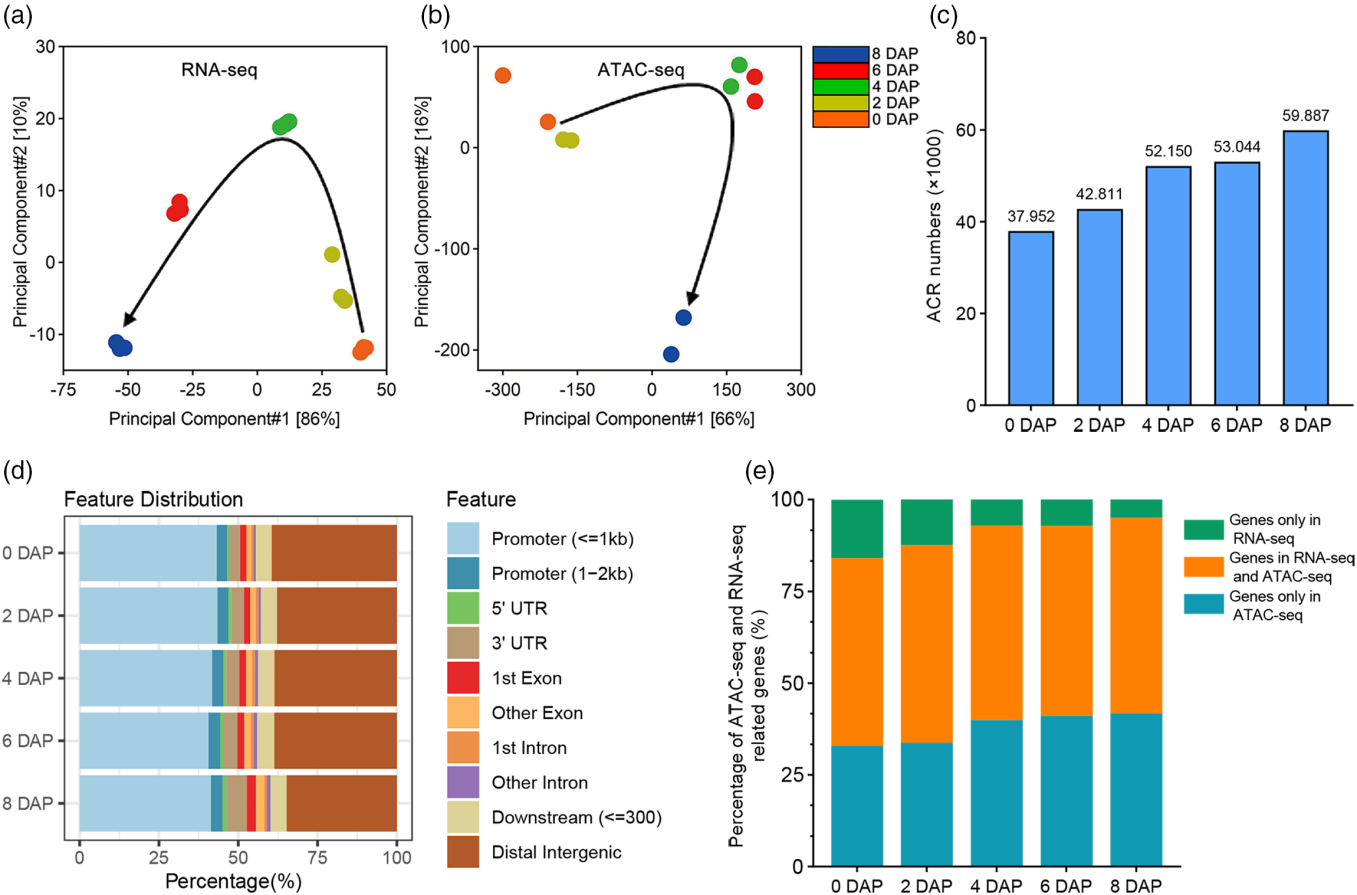
Chromatin accessibility overview during early seed development in maize. (a) and (b) Principal‐components plots of RNA‐seq and ATAC‐seq data, respectively. The black arrows indicate developmental trajectories. Each dot represents one sample. (c) Number of high‐quality ACRs screened by IDR (IDR <0.05) in maize nucellus at different stages. (d) Distribution of ACRs across the genome of maize nucellus at different stages. (e) Overview of expressed genes (TPM >2) and open chromatin‐related genes.

To investigate dynamic chromatin accessibility during early maize seed development, we first identified ACRs in maize (*Zea mays*) inbred line B73 nucellus (embryo sac included, pericarp removed) using ATAC‐Seq (Buenrostro et al., 2013; Lu et al., 2017). In parallel, we performed transcriptome data analysis using the same tissues to explore the impact of chromatin accessibility on gene expression. The ATAC‐seq and RNA‐seq data had two and three biological replicates, respectively, and the replicate samples showed high correlations (Figure 1a,b and Figure S1). We identified a range of 37 952 to 59 887 high‐quality ACRs in the nucellus of maize at 0, 2, 4, 6, and 8 DAP using ATAC‐seq (Figure 1c and Additional file 2: Table S3). The total length of these ACRs ranged from 19.87 to 38.62 megabases (Mb) (Figure S5a). The size of ACRs varies at different DAPs, with lengths mainly around 370 bp from 0 DAP to 6 DAP and around 450 bp at 8 DAP (Figure S3a). ACRs were enriched at transcription start and end sites (TSS, TES), especially with higher signals at TSS (Figure S2). Based on ACR location, we further divided all the ACRs into four sets: ACRs within 2 kb upstream of the TSS (promoter regions, pACRs), ACRs within 0.3 kb downstream of the TES (downstream regions), ACRs within Introns, Exons, and UTR (gene body), and other ACRs (distal intergenic, dACRs). Our results showed that most ACRs were located in the promoter regions, followed by the distal intergenic regions (Figure 1d).

After double fertilization, the structure of maize seed becomes progressively more complex, especially at 8 DAP, almost all cell types appear, while the number and total length of chromatin opening also reach their maximum (Figure 1c and Figure S5a). The pattern of chromatin opening was found to be similar between 0 DAP and 2 DAP, as well as between 4 DAP and 6 DAP (Figure 1b). The trends of chromatin opening and gene expression did not perfectly align, with chromatin opening being more consistent than gene expression (Figure 1a,b). The proportion of genes with open chromatin increased from 0 DAP to 8 DAP (Figure 1e). These results indicate that chromatin opening is constantly changing with maize seed development, showing an overall trend of enhancement, and opening changes are more stable than gene transcription.

### Impact of chromatin accessibility in promoter regions on gene expression

**Figure 2:**
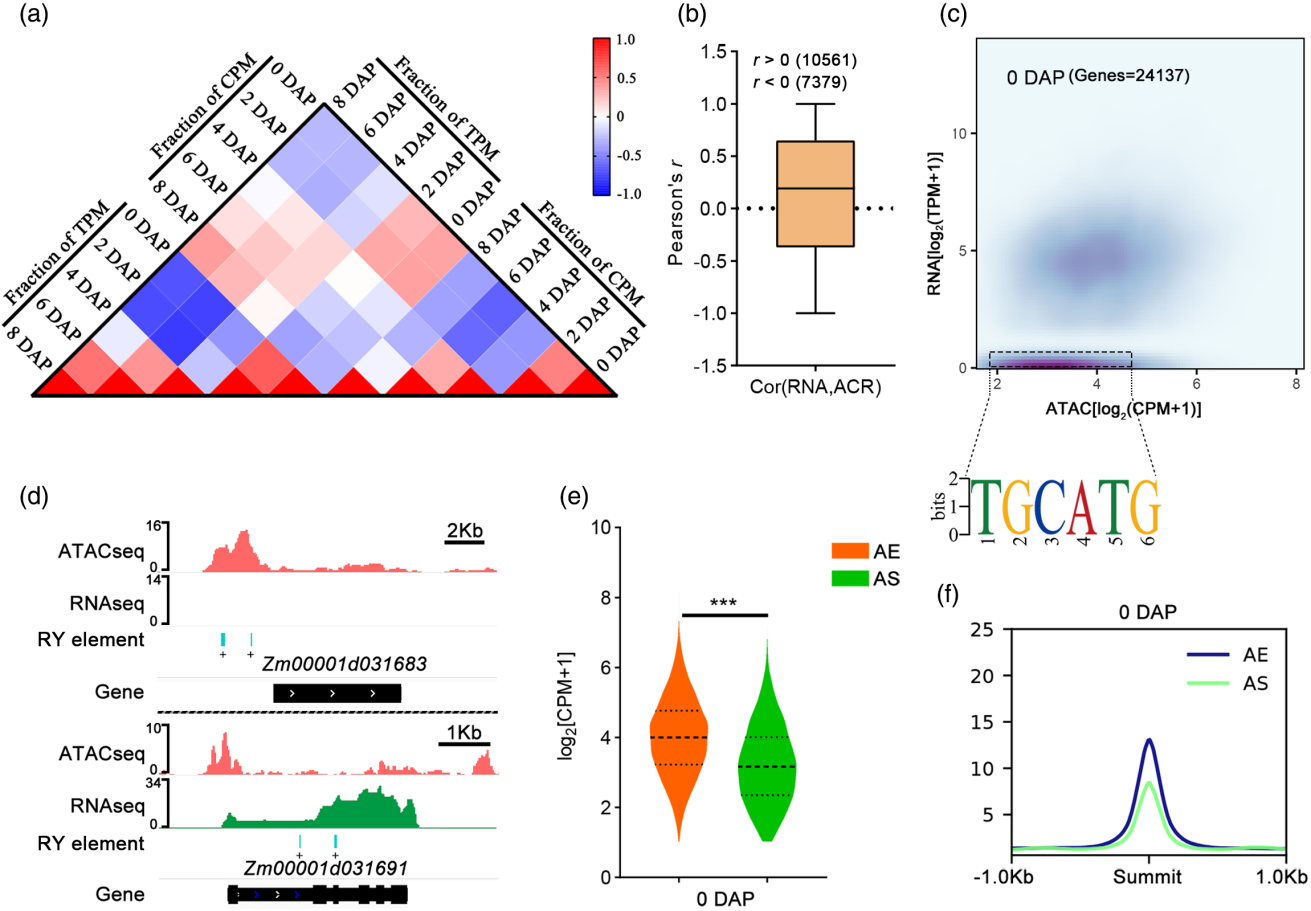
Correlation between chromatin accessibility of promoter regions and gene expression. (a) Heatmap of Spearman correlation between chromatin accessibility (pACR) and associated gene's expression, the gene's CPM (Counts per Million, pACRs), and TPM (Transcripts Per Kilobase of exon model per Million mapped reads) to calculate the proportion of each time point relative to the sum. (b) Boxplots of Pearson's *r* between pACR accessibility scores (CPM) and gene expression values (TPM). (c) Density scatterplot of pACR accessibility (*x* axis), and RNA expression (*y* axis) for each gene at 0 DAP. The *de novo* motifs enriched in pACRs of accessible/silenced genes. (d) The gene's chromatin accessibility (red) and RNA expression (green) which pACR contain/no RY element (cyan). (e) Violin plot showing that the AE (accessible/expressed) pACR accessibility scores are significantly higher than AS (accessible/silenced) pACR accessibility scores at 0 DAP. Statistical significance of the difference was determined by Student's unpaired *t* test.***P < 0.001; **P < 0.01; *P < 0.05. (f) Profile plot of AE and AS at 0 DAP.

Chromatin accessibility at promoter region is a prerequisite for transcription. To evaluate the relationship between pACR accessibility and gene expression, we performed a Spearman correlation analysis of dynamic changes in gene expression and pACR accessibility. A total of 17 940 genes' pACR scores and RNA expression values for analysis. We used Pearson's correlation coefficient to analyze the relationship between pACR and RNA expression. The results showed that 10 561 pACR‐gene pairs had positive correlations (*r* > 0) and 7379 had negative correlations (*r* < 0). In negatively correlated pairs, gene expression mainly decreased from 0 DAP to 8 DAP, while in positively correlated pairs, gene expression increased (Figure S4c,d). We identified 2166 pACR‐gene pairs with p‐values less than 0.05, of which 1586 were positively correlated and 580 were negatively correlated (Additional file 2: Table S2a). During early maize seed development, the accessibility of pACRs was mainly positively correlated with the expression of corresponding genes (Figure 2a,b; Figure S3c). Chromatin accessibility is not only positively correlated with gene expression but also negatively. In our dataset, there were also pACR‐gene pairs whose gene expression was negatively correlated with pACR accessibility (Figure 2b; Figure S3d). Despite the association between gene expression and chromatin accessibility, we observed a series of accessible/silenced genes (Figure 2c; Figure S4b). The accessibility of pACRs for genes that were both accessible and expressed was significantly higher compared to those that were accessible but silenced (Figure 2e,f; Figure S3e, S4a). We used MEME discovers motifs that were enriched in the accessible/silenced relative to the accessible/expressed, including a TGCATG motif (*E*‐value < 8.7e‐205, 36.2% of ARCs, 1872/5165) termed Sph/RY element (Sasnauskas et al., 2018; Suzuki et al., 1997). In Arabidopsis, B3 domain genes bind to the RY motif and recruit PRC2 to silence gene expression. In rice, Sph/RY motifs also recruit repressive complexes that inhibit gene expression through histone deacetylation or H3K27me3 methylation (Guo et al., 2013; Xie et al., 2018). The precise spatiotemporal regulation of gene expression by PRC2 is crucial for normal endosperm and embryo development (Xu & Zhang, 2023; Yuan et al., 2021). Thus, when the Sph/RY element was present in ACR, it could repress gene expression through the PRC (Figure 2d). The observation of a series of accessible/silenced genes despite the association between gene expression and chromatin accessibility highlights the complexity of the regulation of gene expression in maize seed development. In conclusion, these results suggest that the accessibility of pACRs in maize seed development can be seen as an indicator, to some extent, of transcription levels. Furthermore, the accessible polycomb response elements (PREs) appear to regulate gene silencing through their interaction with the polycomb repression complex (PRC) in maize seeds.

### Open chromatin as a precondition for gene expression in seed development

Not all alterations in transcription were accompanied by changes in pACRs accessibility. In our dataset, approximately 31% of the accessible/silenced associated genes were silenced in all periods, and additionally 69% of the genes had an accessible/silenced to accessible/expressed shift (Figure S5b). Gene Ontology (GO) analysis was used to further explore the role of shift genes in early seed development. The shift from accessible/silenced to accessible/expressed genes at ‘0 DAP to 2 DAP’ and ‘4 DAP to 6 DAP’ was significantly enriched in growth‐related and metabolism‐related pathways, respectively (Figure S5d,e). After the shift, changes in ACRs were predominantly positively correlated with changes in gene expression during these developmental stages (Figure S5f,g). The establishment of auxin polarity at 2 DAP activated cell division and at 6 DAP, endosperm cells were metabolically active and began to differentiate. Such as the auxin transport gene *ZmPIN8* and the aleurone cell fate specification and cell differentiation TFs *ZmNKD1* all underwent a shift (Figure S5c). Furthermore, 17 109 genes exhibited closed chromatin in the promoter region, and none showed RNA expression during the period from 0 DAP to 8 DAP. GO analysis revealed significant enrichment primarily in DNA repair, photosynthesis, and response to wounding, among other pathways (Figure S6a). Based on the pACR and transcriptome data, our results suggest that chromatin accessibility may provide a precondition for the rapid expression of related genes during seed development.

### Chromatin accessibility and gene expression dynamics during seed development

In eukaryotic genomes, DNA is compactly packaged in the nucleus by wrapping around histones approximately 1.75 times to form nucleosomes, which constitute the fundamental unit of chromatin (Vaquero et al., 2003). Throughout the lifecycle of plants, gene expression is regulated dynamically by *trans*‐acting factors and CREs that require chromatin to be in an open state (Schmitz et al., 2022). Here, we combined gene expression with chromatin accessibility of pACRs to investigate how chromatin accessibility is involved in seed development.

Firstly, at the RNA level, we screened genes with expression (TPM >2) and subsequently extracted genes that showed significant differences in expression (|log_2_FC| > 1 & *P*adj < 0.05) between at least two periods. A total of 7058 genes were screened and divided into five clusters according to their transcription; the clusters 1, 2, 3, 4, and 5 correspond to 0, 2, 4, 6, and 8 DAP, respectively (Figure S7a). The genes in Cluster 1 (2337 genes) were highly expressed in unpollinated nucellus tissues and related to plastid morphogenesis (Figure S7b). The high expression of these genes in unpollinated nucellus tissues might be linked to the storage of nutrients. Interestingly, several known cell cycle‐related regulatory genes (*RBR3* and *ZmSMR4*) (Li et al., 2019; Sabelli et al., 2005) were highly expressed at 0 DAP, indicating that the cell cycle of unpollinated nucellus tissue was arrested, which prevents indefinite growth (Figure S7a). One thousand one hundred and seventy‐one genes (Cluster 2) were highly expressed in the stage of endosperm cellularization, and these genes were predominantly enriched in the cell division pathway. This result suggests that the endosperm undergoes cellularization after 48 h of double fertilization, and auxin may play an important role in this process (Figure S7b). Subsequently, endosperm cells begin to be differentiated, cell functions become specialized, and genes related to endosperm storage materials (storage protein synthesis: *O7*, *O11*, *Pdk2*, *ZmAFL4*; starch synthesis: *ZmNAC128*, *ZmNAC130*, *Ae1*) (Feng et al., 2018; Grimault et al., 2015; Lappe et al., 2018; Miclaus et al., 2011; Stinard et al., 1993; Zhang et al., 2019) show higher expression (Figure S7a).

**Figure 3:**
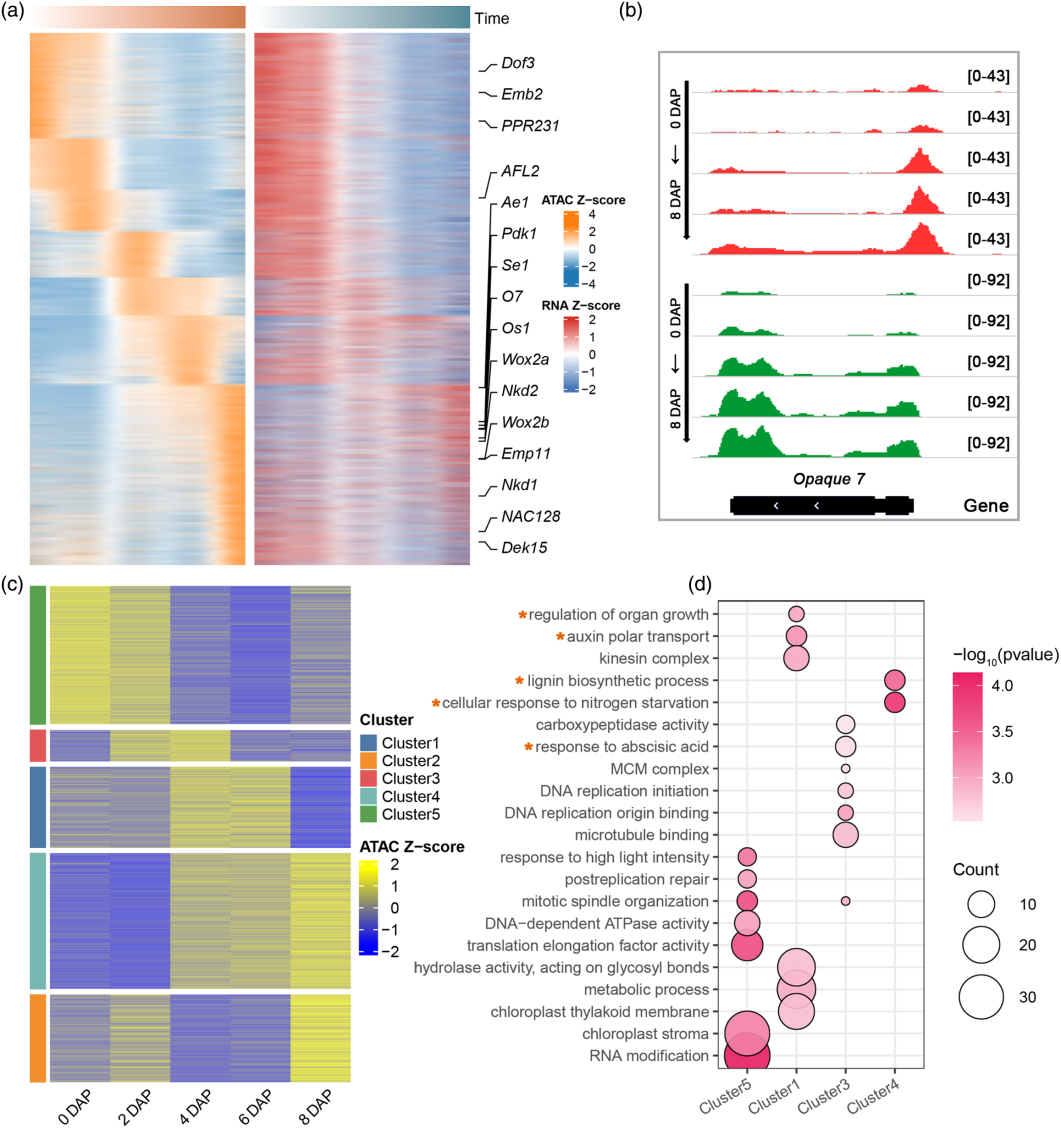
Chromatin accessibility of time‐course pACRs and associated RNA expression profile during seed development. (a) Heatmap of time‐course pACR signals and corresponding gene expression levels. The right side of the heatmap highlights known genes involved in maize seed development. (b) The ATAC‐seq (red) and RNA‐seq (green) profiles of the time‐course pACR for *Opaque7*. (c)Cluster heatmap of time‐course pACRs, divided into five clusters. (d) Gene Ontology (GO) analysis of different clusters (FDR <0.05). Orange asterisks indicate pathways that differ from those observed at the RNA level.

At the pACR level, the time‐course pACR scores did not fully align with the gene expression patterns (Figure 3a). We divided 6892 time‐course pACR‐genes into 5 clusters (Figure 3c and Additional file 2: Table S2). During the early stages of seed development (0 DAP and 2 DAP for Cluster 5), specific pACR‐genes were enriched in RNA modification and chloroplast‐related pathways, similar to the RNA level. During the transition from coenocyte to cellularization (2 DAP, 4 DAP for Cluster 3), the time‐specific pACR‐genes were enriched in DNA replication pathways, mitosis‐related pathways, and responses to abscisic acid (ABA) (Figure 3d) At this stage, the DNA replication pathway was similar at the RNA level, but the ABA pathway differs. The ABA‐related pathway may play a role in the early stages of maize endosperm and embryonic development. During the early stage of endosperm differentiation (4 DAP and 6 DAP for Cluster 1), the main enrichments were in hydrolase activity, metabolic processes, auxin polar transport, and regulation of organ growth. However, during the transition from early to late stages of endosperm differentiation (Cluster 4), the ATAC signal gradually increased. These pACR‐genes were enriched in cellular responses to nitrogen starvation and the lignin biosynthetic process, corresponding to nutrient synthesis in endosperm cells and the degradation of nucellus tissue (Figure 3d). In addition to these, cluster 2 did not show significant enrichment in any pathway.

As cellular types increase in complexity, so does gene expression. With the differentiation of endosperm cells, certain genes exhibit increased activity in expression, paralleled by heightened activity in their respective pACRs. The *Opque7* (*O7*) gene encodes an acyl‐CoA synthetase‐like protein crucial for protein bodies, exhibiting a consistent trend in both pACR degree and expression values (Figure 3b). In the later stages of endosperm differentiation, several known genes identified in the time‐course pACR‐genes are associated with specific functions or tissues. For example, *Ae1*, *Pdk1*, *O7*, *Nkd1*, *Nkd2*, *NAC128*, and *Os1* are linked to material storage, while *Wox2a* and *Wox2b* are involved in embryo development (Figure 3a). In addition, further analysis of the significantly enhanced ACRs from 0 DAP to 8 DAP revealed that they were enriched in cell differentiation, fatty acid metabolism, and phagophore assembly pathways, which are related to seed development (Figure S7c,d). These results suggest that during seed development, there is a process of differentiation where various functional and specialized cell types are formed. This process is accompanied by specific pACR chromatin opening events and the subsequent transcriptional activation of corresponding genes, which ultimately drives the specialized cellular functions required for seed development. We identified these specific time‐course pACRs, which could play crucial roles in the development of maize seeds during specific periods.

### Distal chromatin accessibility in maize seeds

The expression of target genes in distal ACRs can be regulated through interactions between chromatin, according to previous studies (De Laat & Duboule, 2013; Peng et al., 2019; Ricci et al., 2019). In our dataset, distal ACRs (dACRs) account for a high proportion of all types of ACRs (Figure 1d). The number of dACRs and genes with both dACR and pACR increased with seed development (Figure S8b,c). Most dACRs were within 100 kb of their nearest gene (Figure S9a). During seed development, the chromatin accessibility of both pACR and dACR in genes showed a mainly positive correlation and their accessible regions were enriched with similar motifs (Figure S8a,d,e). We calculated the co‐ACRs in our dataset. This analysis identified a total of 137 401 highly correlated co‐accessible pairs, of which 109 459 pairs correspond to gene bodies or promoter regions, including 66 782 positively correlated ACR pairs and 42 677 negatively correlated ACR pairs (Additional file 2: Table S4). *KRN4* is a distal enhancer of the *UB3* promoter, and our data showed that there was a positive co‐accessibility between the *UB3* promoter and a downstream region of about 60 kb, which is consistent with previous reports (Du et al., 2020) (Figure S10a).

**Figure 4:**
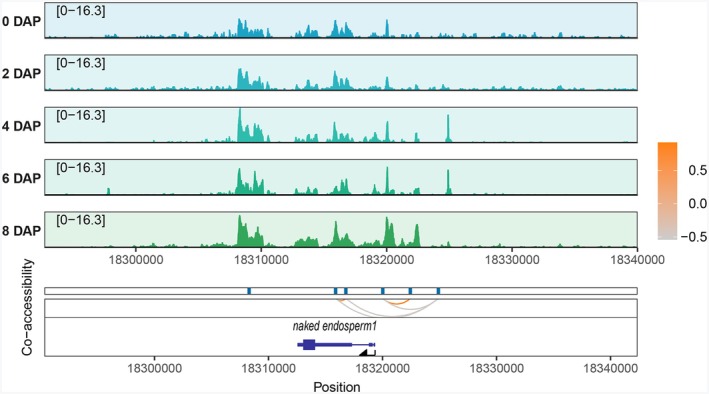
Co‐accessible ACRs during maize seed development. The predicted co‐accessible profile of *NKD1*, with curved lines indicating co‐accessible loops.

Distal ACRs could be utilized to identify additional regulatory sites involved in maize seed development. *ZmNKD1*, a crucial gene for maize endosperm development, exhibits two co‐ACRs: one located approximately 3.4 kb upstream (positive) and the other approximately 6 kb upstream (negative) (Figure 4). Furthermore, a distal ACR located approximately 6 kb upstream of *ZmNKD1* negatively co‐accesses with intron 2 of *ZmNKD1*. In summary, based on the dataset in this study, we can predict and identify additional distal regulatory elements that play a role in maize seed development.

### Transcription factor binding profile during seed development

**Figure 5:**
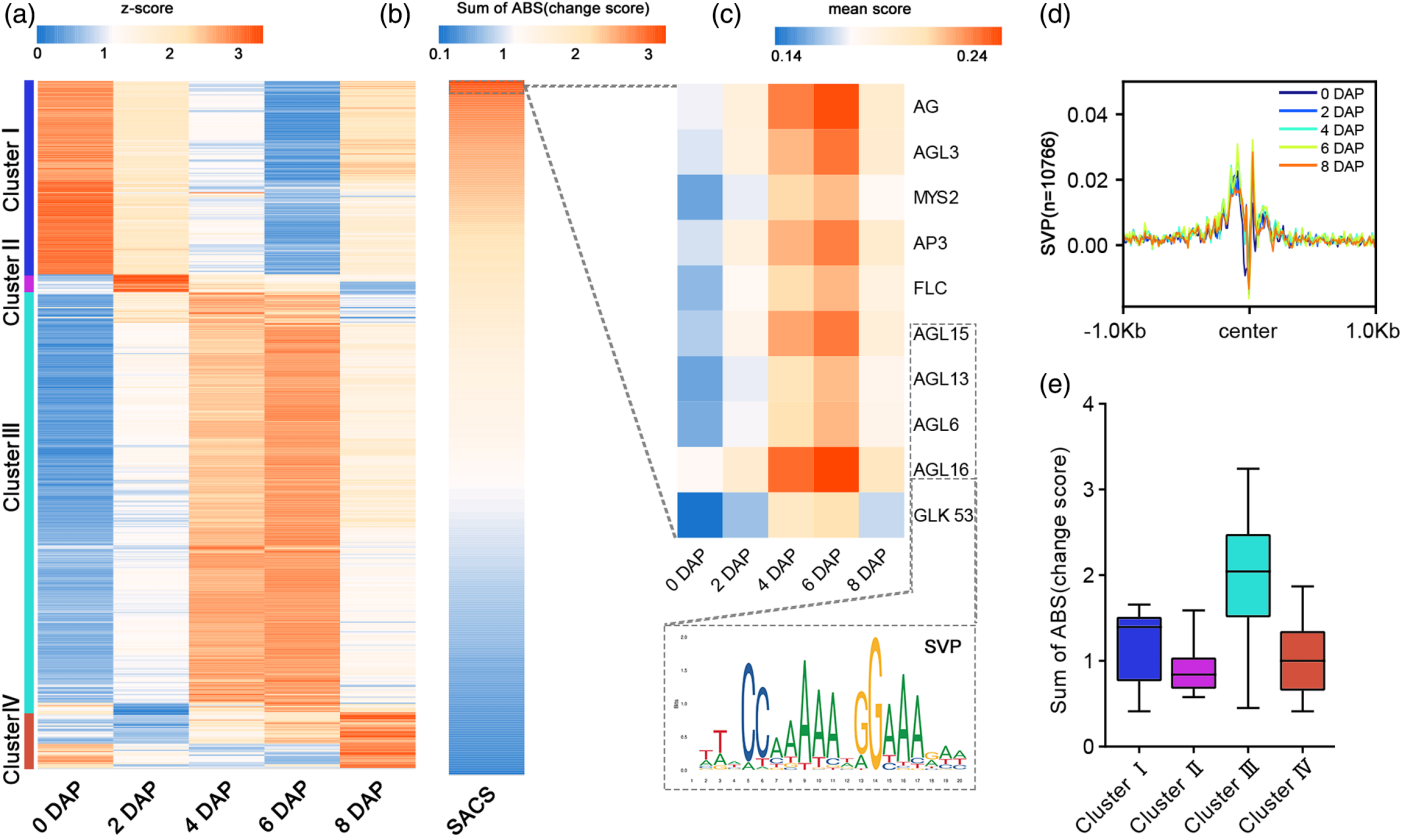
Global changes in transcription factor binding throughout seed development. (a) Heatmap of 568 transcription factor motifs binding score in development seed. (b) The sum of the absolute values of the change scores (SACS) for each motif. (c) Heatmap of binding scores for motifs ranked in the top 10 for SACS values, and the four AGL motifs are similar to the SVP motif. (d) Profile plot of SVP binding strength in different DAP. (e) Boxplots of SACS for four clusters.

Chromatin accessibility and the binding of transcription factors to regions of open chromatin are critical for gene expression. To comprehend the role of transcription factors (TFs) in regulating gene expression during early seed development, we integrated known motifs and our ATAC‐seq data to identify the footprints of TFs. A total of 568 motifs with a change score greater than 0.1, indicating stage specificity during seed development. Clusters I, II, III, and IV showed stronger footprints in unpollinated nucellus, coenocyte stage, early differentiation stage, and late differentiation stage, respectively (Figure 5a). Further classifying the motifs in these clusters, we found that the enriched motif families varied widely among the different clusters. The most abundant variety of motifs was found in cluster III, with cluster I enriched for ERF and bHLH motifs, cluster IV for NAC and bZIP motifs, and cluster II for MYB and GATA motifs (Figure 5e; Figure S11a).

The distribution pattern of motifs was closely associated with the seed development process. We calculated the sum of the absolute values of the change scores (SACS) for motifs in the four clusters and found that the top 10 motifs with SACS values were all located in cluster III. Furthermore, the majority of these motifs belonged to the MIKC‐type MADS family (Figure 5b,c). Members of MADS domain transcription factors usually play an important role in flower development, but it has also been shown that they play a key role in embryo and endosperm development (Steven D. Rounsley et al., 1995, Lee et al., 2013, Heck et al., 1995, Zhang et al., 2024). Motif similarity analysis revealed that members of the MADS family, including AGL6, AGL13, AGL15, and AGL16, shared a consensus motif known as SVP (Figure 5c; Figure S11d). The SVP motif exhibited a strong signal at 4 DAP and 6 DAP, corresponding to the early stages of embryo development (Figure 5c,d) and the expression of SVP also matched footprint signals (Figure S11e). SVP and AGLs both belong to MIKC‐type MADS family, which is mainly located in cluster III. MADS transcription factors may be more critical than other transcription factors in the early stages of embryo and endosperm differentiation. The abundance of transcription factor activities varied significantly among different developmental stages, with the largest number and class observed during the early stage of endosperm cell differentiation (Figure 5a,b). These findings suggest that gene expression regulation is highly complex during the early stage of cell fate determination in maize seeds, with distinct transcription factor families being involved at each stage.

### Differential binding of transcription factors predicts the regulatory network during early embryonic development

**Figure 6:**
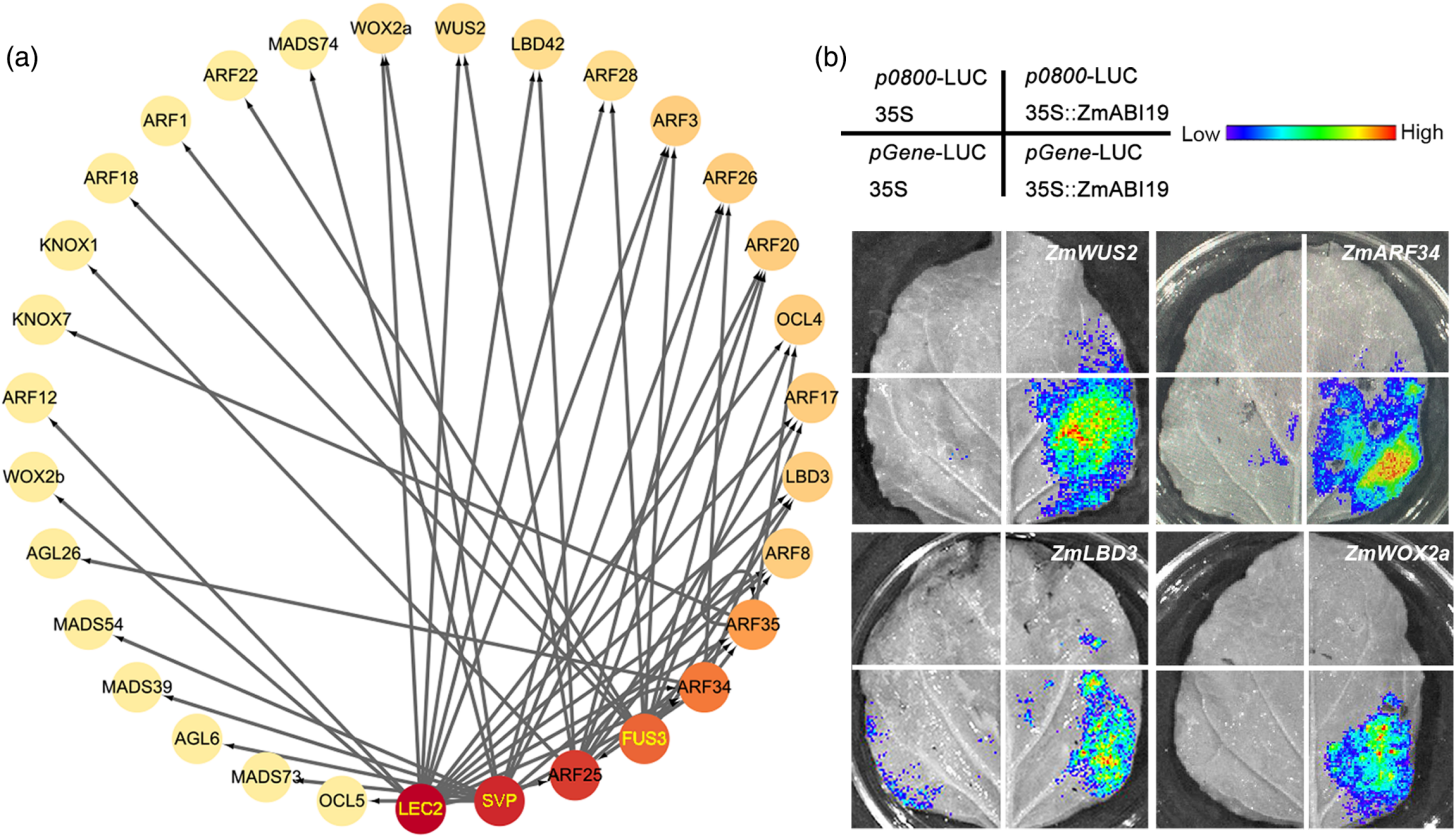
Predicted transcription factor regulatory network for embryonic development based on chromatin accessibility. (a) Transcription factor regulatory network based on LEC2, SVP, FUS3, ARF25, ARF34, and ARF35. Arabidopsis genes are represented in yellow fonts, while maize genes are represented in black fonts. (b) Transient expression assays of *pZmWUS2*‐LUC, *pZmWOX2a*‐LUC, *pZmARF34*‐LUC, and *pZmLBD3*‐LUC in tobacco leaves expressing 35S::ZmABI19. Empty vectors *p0800*‐LUC and 35S were included as controls.

In addition to SVP‐like motifs, the B3 domain transcription factors LEC2 and FUS3, which are known to play a role in embryonic development (Roscoe et al., 2019; Stone et al., 2001; Tsuchiya et al., 2004), were found to be present in cluster III (Figure S11c). Furthermore, ARFs also play a significant role in maize embryo morphogenesis (Crawford et al., 2015; Verma et al., 2022). The binding sites of the predicted ARFs were found to be associated with the auxin pathway, indicating the reliability of our analysis (Figure S11b). Thus, we constructed a proposed transcription factor regulatory network centered on SVP, LEC2, and FUS3 (from Arabidopsis) as well as ARF25, ARF34, and ARF35 (from maize) to predict the mechanism of maize embryogenesis (Figure 6a). In Arabidopsis, it has been shown that the transcription factor AtLEC2 promotes somatic embryogenesis by directly activating the genes *AtWOX2* and *AtWOX3* (Wang et al., 2020). Our data indicate that LEC2 binding motif was found in the promoters of *ZmWOX2a* and *ZmWOX2b*, as well as in *ZmWUS2*, *ZmARFs*, and *ZmLBDs*. In Arabidopsis, *WUSCHEL* (*WUS*) is expressed in the 16‐cell pro‐embryo during zygotic embryogenesis, preceding the development of stem cells and the embryonic shoot (Mayer et al., 1998). The accumulation of auxin is crucial for apical formation during embryogenesis (Verma et al., 2021). In maize, following double fertilization, the zygote gradually develops into a mature embryo, during which apical stem cell pools are established.

To identify the maize homolog of *AtLEC2*, we searched the B3 domain of the maize genome and retrieved a total of 101 genes (*E*‐value <1e‐5). Then, we constructed a phylogenetic tree based on the full‐length protein sequences of the 101 B3 domain genes and AtLEC2. The B3 domain transcription factors VP1 and ZmABI19 showed homology to AtLEC2 (Figure S12b). *ZmABI19* has been reported to play a crucial role in seed development, specifically in the synthesis of storage materials in the endosperm and embryo. In addition, *zmabi19* exhibited defective embryos with no root meristem and abnormal shoot meristem (Yang et al., 2021). And *ZmABI19* showed predominant expression in the embryo at 8DAP, the data from (Zhan et al., 2015) (Figure S12a). Although ABI19 expression decreased during the early stages of seed development, it increased significantly at 8 DAP during embryogenesis and differentiation (Figure S11f). So, we hypothesized that *ZmABI19* functions similarly to *AtLEC2* in maize embryo development. Luciferase assays showed that ZmABI19 was able to bind to the promoters of *ZmWUS2*, *ZmARF35*, *ZmWOX2a*, and *ZmLBD3* (Figure 6b). These findings suggest that ZmABI19 may play an important role in establishing embryonic polarity during early development in maize. Taken as a whole, our analysis of chromatin accessibility profiles enabled us to construct a regulatory network of transcription factors during seed development.

### Accessible chromatin regions are hotspots of phenotypic variation

**Figure 7:**
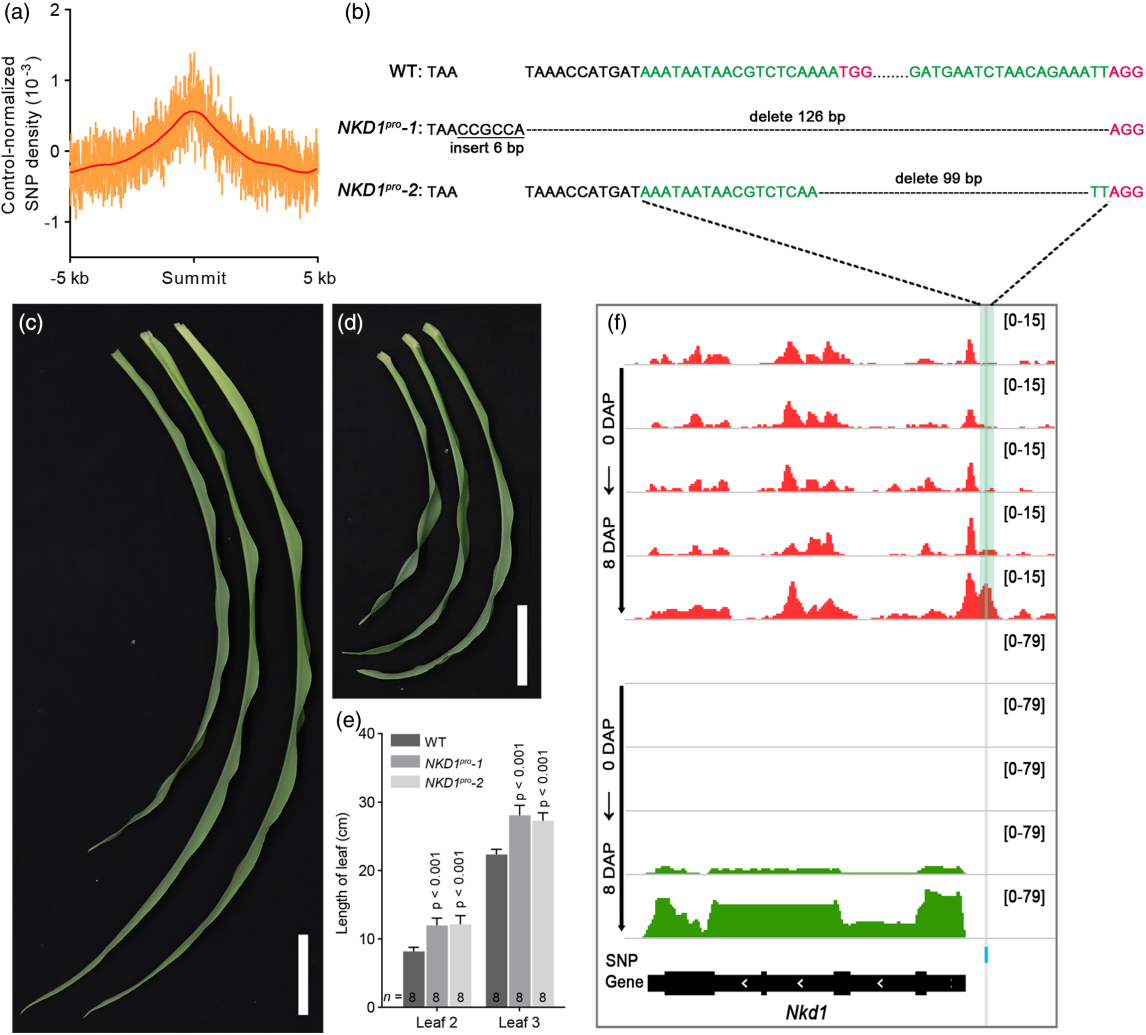
Phenotypically associated genetic variants are enriched in accessible chromatin regions. (a) Relative enrichment of phenotype‐associated SNPs for 5‐kb regions flanking ACR summits. (b) The CRISPR‐*Cas9* editing type of NKD1‐pACR, with the green region indicating the guide sequence and the red region indicating the PAM site. (c) and (d) show the leaf length of leaf 3 and leaf 2, respectively. From left to right, the samples are WT, *NKD1* ^*pro*^ *‐1*, and *NKD1* ^*pro*^ *‐2*. Scale bar = 3 cm. (e) Bar plot showing the leaf lengths of leaf 2 and leaf 3. Statistical significance between the WT and *NKD1* ^*pro*^ groups was assessed using a *t*‐test. (*n* denotes the number of independent biological replicates). (f) The ATAC‐seq (red) and RNA‐seq (red) profiles of *NKD1* were shown. The gray line and blue bar indicated the SNP site, while the light green region represented the CRISPR‐*Cas9* edited region.

The genetic variation of maize is abundant in nature, which leads to its wide distribution and diverse phenotypes. Understanding the relationship between phenotype and genetic variation is important for improving maize yield and quality. Genome‐wide association study (GWAS) has identified many Single nucleotide polymorphisms (SNPs) associated with agronomic traits in maize (Yang et al., 2014; Zeng et al., 2022). Many studies have shown that variation in key amino acids can lead to phenotypic variation. In addition to variation in genic regions, many variants located in *cis*‐regulatory modules (CRMs) are also important sources of phenotypic variation (Long et al., 2016; Lu et al., 2019; Schmitz et al., 2022; Wallace et al., 2014). In our dataset, DNA sequence diversity was significantly reduced at regions enriched with CRMs, and the binding sites for transcription factors were more conserved compared to the flanking sequences (Figure 7a; Figure S13a). In terms of diversity within species, trait‐associated SNPs were more likely in ACRs (Figure S13b). The majority (77%) of trait‐associated SNPs were found outside of gene body regions, highlighting the significant role of gene expression regulation in phenotypic variation (Figure S13c).

We identified a trait‐associated SNP that was located in the pACR of *NKD1*. Interestingly, this SNP was associated with leaf length. We used the CRISPR‐*cas9* system to edit the pACR of *NKD1*, which contains the trait‐associated SNP (Figure 7f). Two types of mutations were obtained using CRISPR‐*Cas9*. *NKD1* ^*pro*^ *‐1* had a 6 bp insertion and a 126 bp deletion, while *NKD1* ^*pro*^ *‐2* had a 99 bp deletion (Figure 7b). The seeds of the two mutants exhibited no significant phenotypic changes (Figure S13e,f), consistent with the findings reported by Wu (Wu et al., 2024). However, the leaf lengths of the mutants differed from those of the WT. After 14 days of growth, the lengths of leaves 2 and 3 in the mutants were significantly longer than WT (Figure 7c–e; Figure S13g–i). In addition, the expression of *NKD1* in leaf 2 and leaf 3 showed changes, with both being higher in the mutants (Figure S13j,k). In summary, editing ACRs at potential phenotype‐associated loci could lead to corresponding phenotypic changes through the alteration of gene expression. Loci associated with phenotypes that are located within ACRs are more likely to contribute to those phenotypes.

Transposable elements (TEs) have contributed greatly to enriching the maize genome (Catlin & Josephs, 2022; Stitzer et al., 2021). TEs can influence gene expression through several different mechanisms, including disrupting CRMs, creating CRMs, and spreading methylation modifications to CRMs (Hirsch & Springer, 2017; Schmitz et al., 2022). By analyzing the overlap between TEs and ACRs, we found that over than 90% of the TEs were located in nongenic regions, indicating that TEs primarily influence gene expression by modulating CRMs (Figure S13d). In conclusion, variations in ACRs are important sources of trait diversity, and ACRs can be prioritized when studying variation in some traits.

## DISCUSSION

Chromatin state plays a vital role in gene expression regulation through its complex pattern of methylated modifications and transcription factor bindings in ACRs. Most ACRs are located in non‐coding regions, and their functions remain largely unexplored in most plant species. Previous research has identified a number of CREs from chromatin accessibility regions, including *tb1* (Studer et al., 2011), *BX1* (Zheng et al., 2015), *KRN4* (Du et al., 2020), *ZmCCT9* (Huang et al., 2018), and *ZmRap2.7* (Salvi et al., 2007), that have been found to influence gene expression. Advancements in the study of maize ACRs have been made in multiple tissues (Dai et al., 2022; Parvathaneni et al., 2020; Vera et al., 2014), but not in the early stages of seed development. Through ATAC‐seq of 0–8 DAP seeds, we identified 37 952 to 59 887 high‐quality ACRs, respectively (Figure 1c). Specific pACRs provide new insights into the biological processes during the early stages of seed development (Figure 3d).

Chromatin accessibility in a gene's promoter region is crucial for regulating gene transcription. There was a subset of pACRs‐related genes that were not expressed, and these pACRs were found to be enriched for repressive motifs of gene expression (Figure 2c,d). We hypothesized that these ACRs (accessible/silenced) would recruit repressive modifiers to prevent the expression of the corresponding genes. ACRs may also function as silencers to suppress gene expression, highlighting their dual role in both activating and repressing transcription during seed development. To confirm this hypothesis, further investigations will be necessary, involving biochemical and relevant mutant studies. Most correlations between pACR chromatin accessibility and gene expression were positive, though some showed negative correlations (Figure 2b). We found that chromatin accessibility is relatively more stable than gene expression during early seed development (Figure 1a,b), perhaps because gene transcription is also regulated by *trans*‐acting factors. Although the maximum chromatin accessibility and maximum gene expression did not occur exactly at the same stage of seed development, the degree of overlap between the two increased as seed development (Figure S3b). Changes in ACRs within promoter regions are likely the most direct indicators of how ACRs regulate plant development, showing a strong correlation with seed development (Figure S7d). This correlation may arise from the increasing specialization of cells during seed development, which demands the precise expression of specific genes. Consequently, the chromatin accessibility of pACRs becomes more closely linked to gene expression as seed develop.

One of the most important functions of ACR is to bind transcription factors to regulate gene expression. Chromatin accessibility region analysis (footprinting) reveals the binding profiles of transcription factors (Gusmao et al., 2014; Gusmao et al., 2016). Through footprinting analysis, we discovered that ZmABI19 was capable of binding to the open promoters of *ZmWUS2*, *ZmWOX2a*, *ZmARF25*, and *ZmLBD3*. It has been demonstrated that *ZmWUS2* and *ZmWOX2a* can enhance the transformation efficiency of immature maize embryos (Che et al., 2022; Lowe et al., 2018; McFarland et al., 2023). *ZmABI19* and other downstream genes may influence transformation efficiency, but further experiments are needed to confirm this connection. ACRs contain numerous CREs, which can be analyzed through enrichment analysis to identify corresponding *trans*‐acting factors, enabling the construction of gene regulatory networks. Although we identified different transcription factors enriched at various stages of early seed development, some tissue‐specific transcription factor binding footprints may be overwhelmed because the sequencing was performed on the whole seed. Furthermore, incomplete motif data significantly restricts transcription factor binding analysis. The application of single‐cell ATAC‐seq technology and the development of transcription factor binding motifs will solve these problems (Farmer et al., 2021; Marand et al., 2021; Sinha et al., 2021). DNA affinity purification sequencing (DAP‐seq) is a powerful tool for detecting transcription factor binding sites (Bartlett et al., 2017), but its accuracy is limited by the lack of an actual chromatin open state in vivo. The genome‐wide ACRs obtained by ATAC‐seq can greatly compensate for this drawback.

Maize diversity is strongly linked to variation in CRMs, with trait‐associated variations enriched in ACRs (Figure S13b). The efficiency of marker‐assisted selection (MAS) can be improved by the analysis of markers in ACRs (Parvathaneni et al., 2020). *NKD1* is known to be involved in seed endosperm development, and generally, a single mutant of *NKD1* does not cause obvious phenotypic changes in seeds (Gontarek et al., 2016; Wu et al., 2024). By combining our chromatin accessibility data with association maps of multiple traits (Wallace et al., 2014), we identified a SNP in the promoter of *NKD1* associated with leaf length, which we then verified using CRISPR‐*Cas9*. In crops with large genomes, regulatory regions represent only a small fraction of the genome. Precisely identifying the number and locations of ACRs can provide valuable insights for understanding and improving key agronomic traits.

In conclusion, the ATAC‐seq and RNA‐seq datasets in this study provide a new perspective for the study of early maize seed development. Further genetic and biochemical experiments are essential to verify that chromatin accessibility regulates maize seed development.

## EXPERIMENTAL PROCEDURES

### Plant material and growth conditions

The maize (*Zea mays*) inbred line B73 was grown in the field in Apr of 2020 in Taian, China, and it was pollinated in Jun. All the individual plants were self‐pollinated at the same time. The nucellus (embryo sac included, pericarp removed) was collected by manual dissection of the seeds. The nucellus was frozen immediately with liquid nitrogen and stored at −80°C before the experiment. The ATAC‐seq and RNA‐seq experiments were performed with 2 or 3 biological replicates at each time point, each replicate consisting of samples from at least 3 individual plants.

### Preparation and sequencing of ATAC‐seq libraries

ATAC‐seq was performed as described previously and partially modified (Buenrostro et al., 2013; Lu et al., 2017). For each replicate, at least 10 nucellus were ground in liquid nitrogen and then transferred to 1 mL lysis buffer (25 mM Tris–HCl pH 7.6, 10 mM magnesium chloride, 0.44 M Sucrose, 0.1% Triton‐X‐100, 10 mM 2‐mercaptoethanol, 2 mM spermine, 0.1 μM phenylmethylsulfonyl fluoride and 1 X cocktail) for nuclear extraction. The nuclear extract was filtered through a 40‐μm cell strainer. Nuclei were stained with DAPI and their status was examined under a fluorescence microscope.

About 50 000 nuclei were resuspended from 50 μL of the reaction solution (10 μL 5 X TTBL, 5 μL TTE Mix V50 and 35 μL ddH_2_O). Incubate at 37°C for 30 min and mix gently every 5 min. The integration products were purified using a Qiagen QIAquick PCR Purification Kit and then amplified using Vazyme TruePrep DNA Library Prep Kit V2 for Illumina for 11–13 cycles. The number of amplification cycles was determined as described previously (Buenrostro et al., 2013). The amplified library was purified using Vazyme VAHTS DNA Clean Beads. The raw data were generated using Illumina NovaSeq 6000 by Annoroad Gene Tech. (Beijing) Co., Ltd.

### ATAC‐seq raw data processing and alignment

Raw reads were trimmed with Trimmomatic v.0.36 (Bolger et al., 2014). Reads were trimmed for NexteraPE with a maximum of two seed mismatches, a palindrome clip threshold of 30, and a simple clip threshold of 10. Reads shorter than 30 bp were discarded. Trimmed reads were aligned to the *Z. mays* AGPv4 reference genome (Jiao et al., 2017) using Bowtie2 v.2.3.5.1 (Langmead & Salzberg, 2012) with the following parameters: ‘bowtie2 ‐X 1000 ‐‐very‐sensitive ‐‐local. Aligned reads were sorted using SAMtools v.1.15 (Li et al., 2009) and PCR clonal duplicates were removed using Picard version v.2.26.7 (http://broadinstitute.github.io/picard/). Read counts and alignment statistics for all samples were provided in Additional Table S1.

### RNA‐seq raw data processing, alignment and expression quantification

Raw reads were trimmed with Fastp v.0.23.2 (Chen et al., 2018) using default parameters. The clean reads were aligned to the *Z*. *mays* AGPv4 reference genome (Jiao et al., 2017) using HISAT2 v.2.1.0 (Kim et al., 2015). Gene expression values were quantified using featureCounts v.2.0.1 (Liao et al., 2014) (maize annotation version AGPv4.48). DESeq2 v1.30.1 (Love et al., 2014) was used to screen for genes with at least a two‐fold expression change and statistically significant differences in expression (adjusted *P* value cutoff of 0.05 and TPM >2) among all samples. Read counts and alignment statistics for all samples were provided in Additional Table S1.

### Identification of ACRs

Macs2 v.2.2.7.1 (Zhang et al., 2008) was used to find all the ACRs, with the parameter set to “‐‐keep‐dup all.” Then IDR v.2.0.4.2 (Li et al., 2011) was used to identify highly reliable ARCs in the two replicates (soft‐idr‐threshold 0.05). The ACR scores for each sample were calculated by DiffBind v.3.0.15 (Ross‐Innes et al., 2012) with the setting “DBA_SCORE_TMM_READS_EFFECTIVE_CPM.” The location of ACRs was defined by ChIPseeker v.1.26.2 (Yu et al., 2015) with the parameter ‘tssRegion = (−2000,0)’, and ACRs were annotated to their nearest gene. ACRs information for all samples were provided in Additional file 3: Table S3.

### Time‐course and Co‐accessible ACRs analysis

The time‐course‐specific ACRs and co‐accessible ACR pairs were identified using CisDynet v.1.0.0 (Zhu et al., 2023). For time‐course‐specific ACRs, the top_N was set to 2000, and these ACRs were clustered into 5 clusters. For the co‐accessible ACRs analysis, the window size was set to 100 000.

### Motif and GO analysis

Motif enrichment of ACRs was performed using MEME‐ChIP v.5.0.1 (Machanick & Bailey, 2011), and Tomtom (Gupta et al., 2007) was used for motif comparison. GO enrichment analysis was performed using clusterProfiler v.4.4.4 (Wu et al., 2021) with the *Z*. *mays* AGPv4 GO annotation from Gene Ontology (Ashburner et al., 2000). Motif similarity analysis was performed using TOBIAS (Bentsen et al., 2020), focusing on binding sites.

### ATAC‐seq footprinting

TOBIAS was used to analyze the transcription factor footprints of ACRs. Analyses were performed using default parameters. Motifs with at least one absolute value of change score greater than 0.1 will be used in subsequent studies. The sum of the absolute values of the change scores (SACS) for these motifs was also calculated. The transcription factor motif data were obtained from JASPAR (Castro‐Mondragon et al., 2022).

### Heatmap and correlation analysis

The R package pheatmap v.1.0.12 was used to create the heatmap for this paper. Pearson correlation coefficients were calculated using the R package cor.test v.4.0.3. The results of the Pearson correlation coefficient analysis were provided in Additional file 2: Table S2.

### Analysis of SNPs and TEs enrichment within ACRs

The distribution of SNPs and TEs in ACRs was analyzed using BEDTools v.2.30.0 (Quinlan & Hall, 2010). The variation data was obtained from https://plants.ensembl.org/zea_mays, and trait‐associated SNP data was obtained from a previous study (Wallace et al., 2014). TEs data was obtained from MaizeGDB (Portwood 2nd et al., 2019).

## Accession Numbers

The raw data generated from this study have been uploaded to the NCBI SRA (Sequence Read Archive) database and can be downloaded using the accession number PRJNA992627 (Wallace et al., 2014).

## Author Contributions

XYZ is responsible for the distribution of all materials associated with this article. XYZ conceived the project and designed the experiments; XSZ and XYZ provided constructive suggestions on designing experiments and writing manuscripts; GMZ and JJW performed the data analysis with some help from YJZ, CZ, RCR, and YMW; JJW, GMZ, and JL performed the experiments; GMZ and JJW wrote the manuscript. All authors approved the manuscript.

## Conflict of Interest Statement

The authors declare that they have no competing interests.

## Supporting information


**Figure S1.** ATAC‐seq biological repeat correlation analysis.
**Figure S2.** ATAC‐seq signal distribution and changes.
**Figure S3.** Correlation between the accessibility of pACR and gene expression.
**Figure S4.** The relationship between chromatin accessibility and gene expression.
**Figure S5.** Chromatin accessibility and gene expression changes in seed development.
**Figure S6.** Gene ontology analysis of silenced and inaccessible genes.
**Figure S7.** Stage‐specific gene expression from 0 DAP to 8 DAP.
**Figure S8.** The dACR of development seed.
**Figure S9.** The distribution of dACR in the early stages of maize seed development.
**Figure S10.** Co‐accessible analysis of *UB3*.
**Figure S11.** Distribution of transcription factor footprints.
**Figure S12.** LEC2 homology analysis.
**Figure S13.** Variations in chromatin accessible regions.

**Table S1.** Summary of data generated in this study.

**Table S2.** List of Pearson's *r* for pACR and RNA. List of time‐course pACR‐related gene and corresponding clusters.

**Table S3.** List of accessible chromatin regions identified in this study.

**Table S4.** List of predicted co‐accessible ACRs in seed.

## Data Availability

The data that support the findings of this study are openly available in the NCBI Sequence Read Archive (SRA) at https://www.ncbi.nlm.nih.gov/sra, accession number PRJNA992627.
