# Supplementary material for: The chromatin accessibility landscape during early maize seed development
Source: Plant J. 2025 Mar 24;121(6):e70073. doi: 10.1111/tpj.70073 (PMC11932762; doi:10.1111/tpj.70073)
Supplement: Supplementary file 1 — Figure S1. ATAC‐seq biological repeat correlation analysis. Figure S2. ATAC‐seq signal distribution and changes. Figure S3. Correlation between the accessibility of pACR and gene expression. Figure S4. The relationship between chromatin accessibility and gene expression. Figure S5. Chromatin accessibility and gene expression changes in seed development. Figure S6. Gene ontology analysis of silenced and inaccessible genes. Figure S7. Stage‐specific gene expression from 0 DAP to 8 DAP. Figure S8. The dACR of development seed. Figure S9. The distribution of dACR in the early stages of maize seed development. Figure S10. Co‐accessible analysis of UB3. Figure S11. Distribution of transcription factor footprints. Figure S12. LEC2 homology analysis. Figure S13. Variations in chromatin accessible regions. [file TPJ-121-0-s004.pdf]

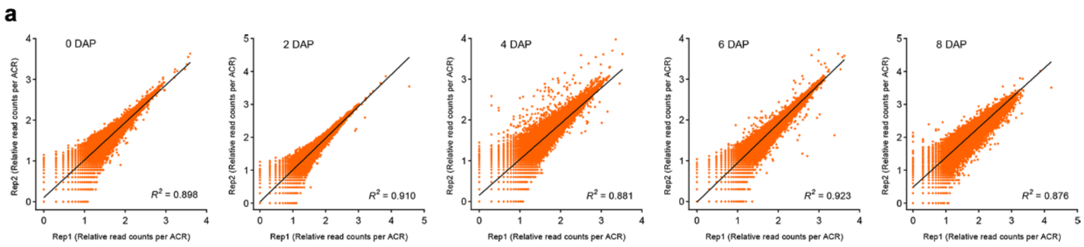

Figure S1. ATAC-seq biological repeat correlation analysis. (a) ATAC-seq reads correlation plot of 0, 2, 4, 6, 8 DAP. R^2: Coefficient correlation, black line: correlation line.

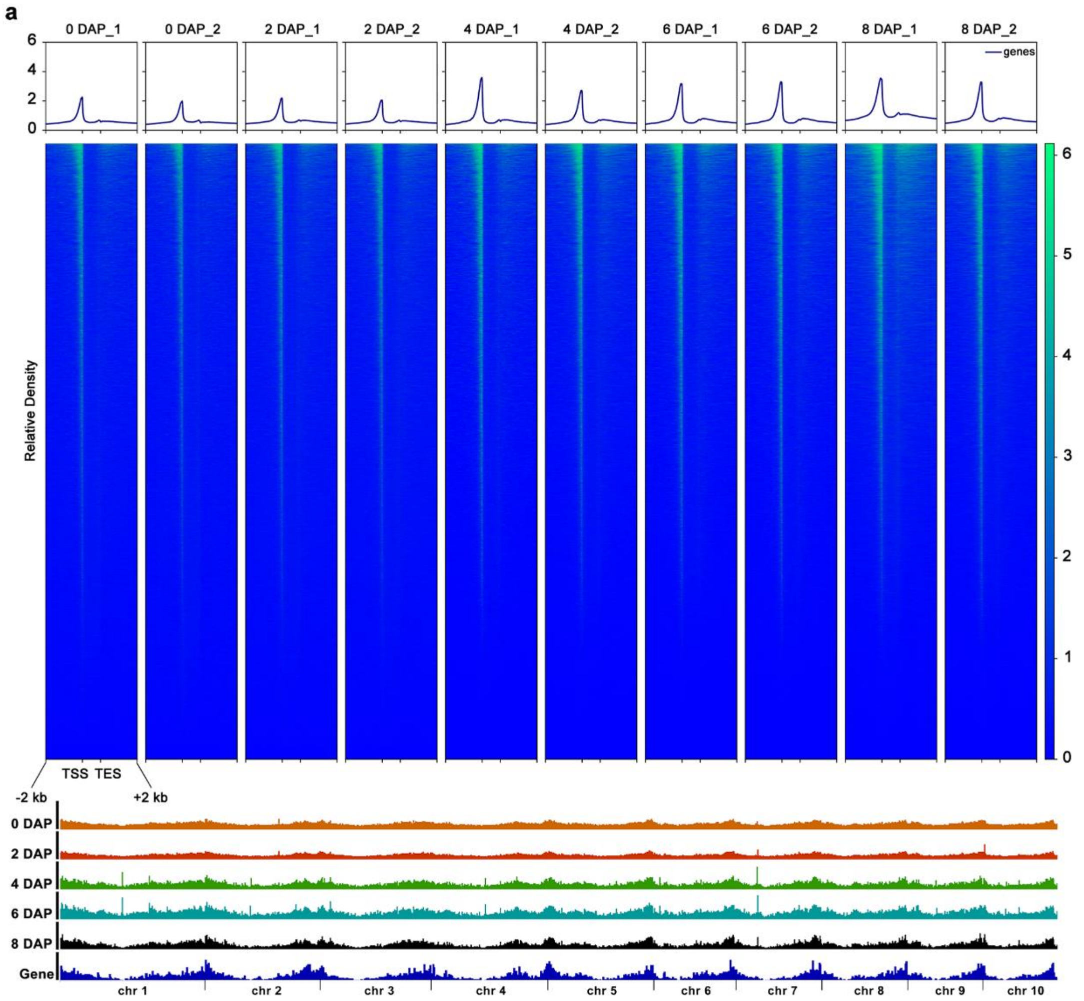

Figure S2. ATAC-seq signal distribution and changes. (a) Heatmap of chromatin opening changes during seed development. 2 kb upstream of the TSS and 2 kb downstream of the TES were plotted. The upper panel shows ACR signals of the gene's upstream and downstream regions up to -2 kb, while the lower panel displays ACR signals across the entire chromosome.

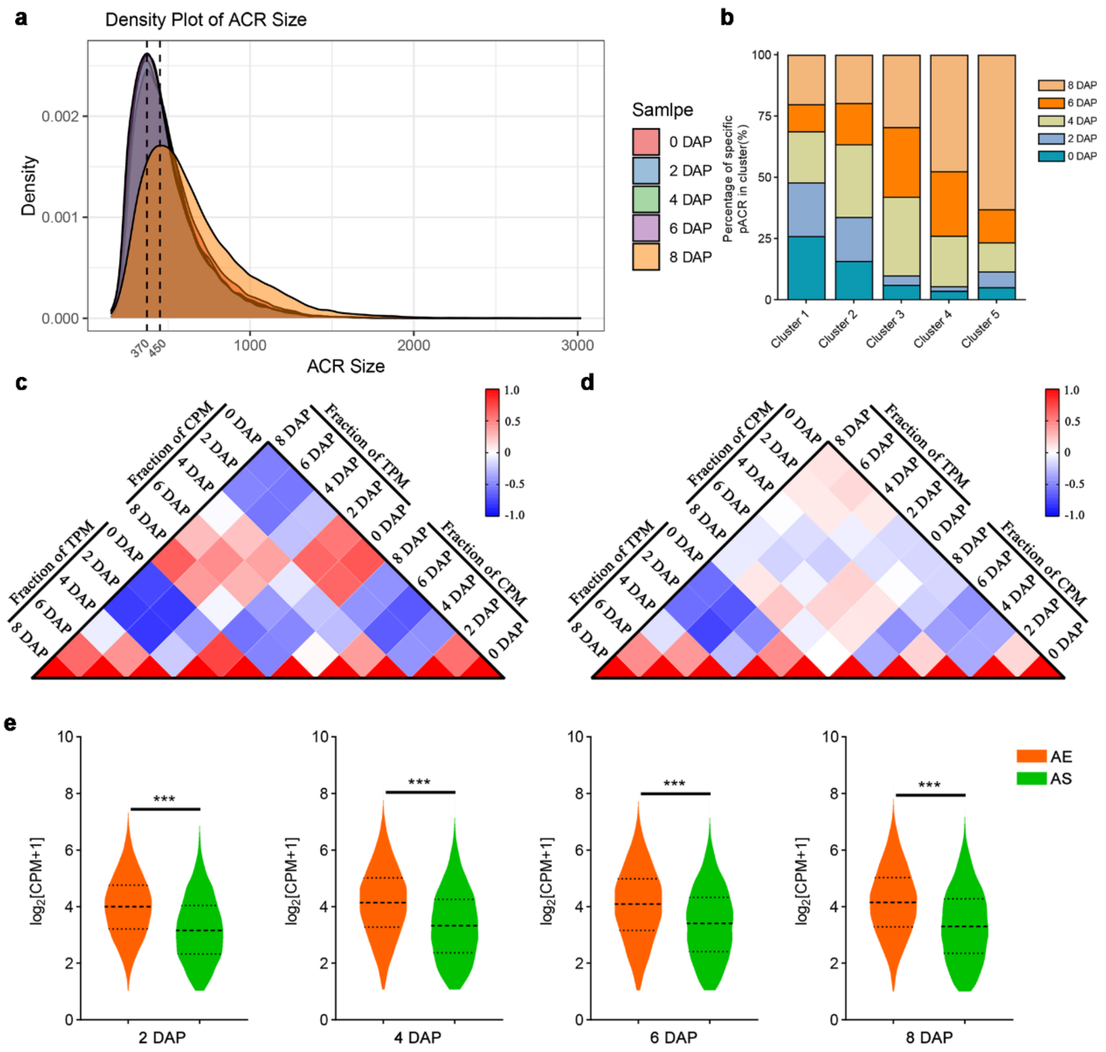

Figure S3. Correlation between accessibility of pACR and gene expression. (a) Density plot of ACR sizes for five samples. **(b)** Percentage of DAPs of highest pACR accessibility in each cluster. **(c)** Heatmap of Spearman correlation between chromatin accessibility (pACR) and associated gene's expression, use the Pearson's r > 0. **(d)** Heatmap of Spearman correlation between chromatin accessibility (pACR) and associated gene's expression, use the Pearson's r < 0. **(e)** Violin plot showing that the AE (accessible/expressed) pACR accessibility scores significantly higher than AS (accessible/silenced) pACR accessibility scores at 2, 4, 6, 8 DAP. Statistical significance of the difference was determined by Student's unpaired t test. ***p < 0.001; **p < 0.01; *p < 0.05.

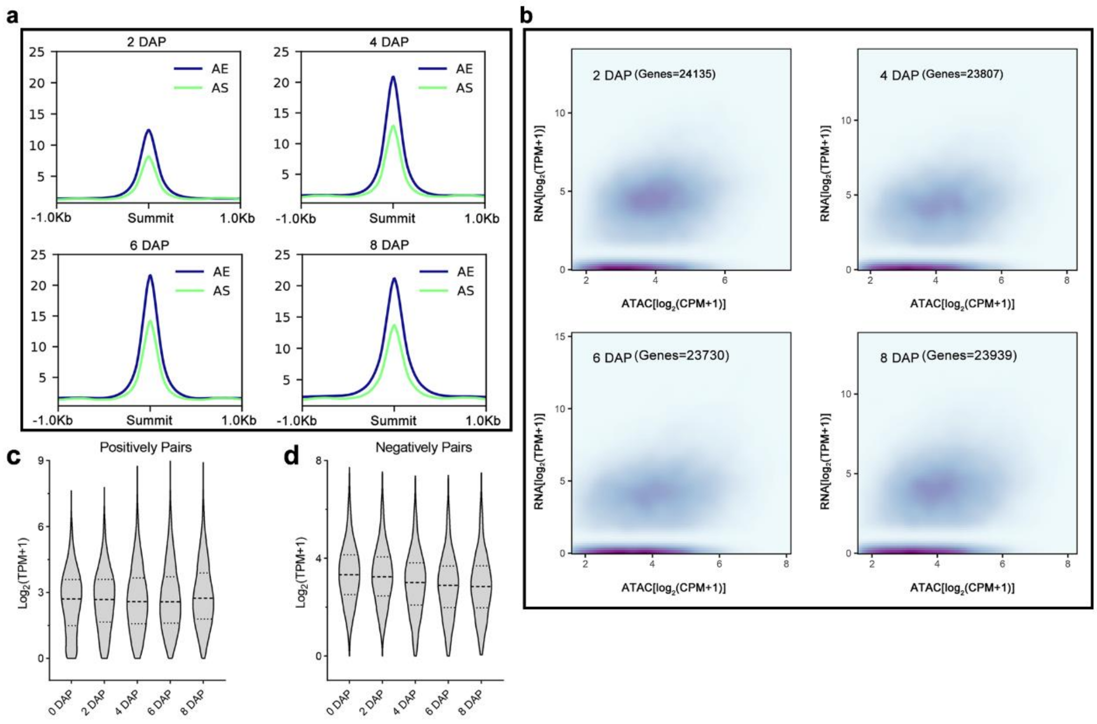

Figure S4. The relationship between chromatin accessibility and gene expression. (a) Profile plot of AE (accessible/expressed) and AS (accessible/silenced) at 2, 4, 6, 8 DAP. **(b)** Density scatterplot of pACR accessibility (x axis) and RNA expression (y axis) for each gene at 2, 4, 6, 8 DAP. **(c)** and **(d)** Gene expression values of positively and negatively correlated pairs.

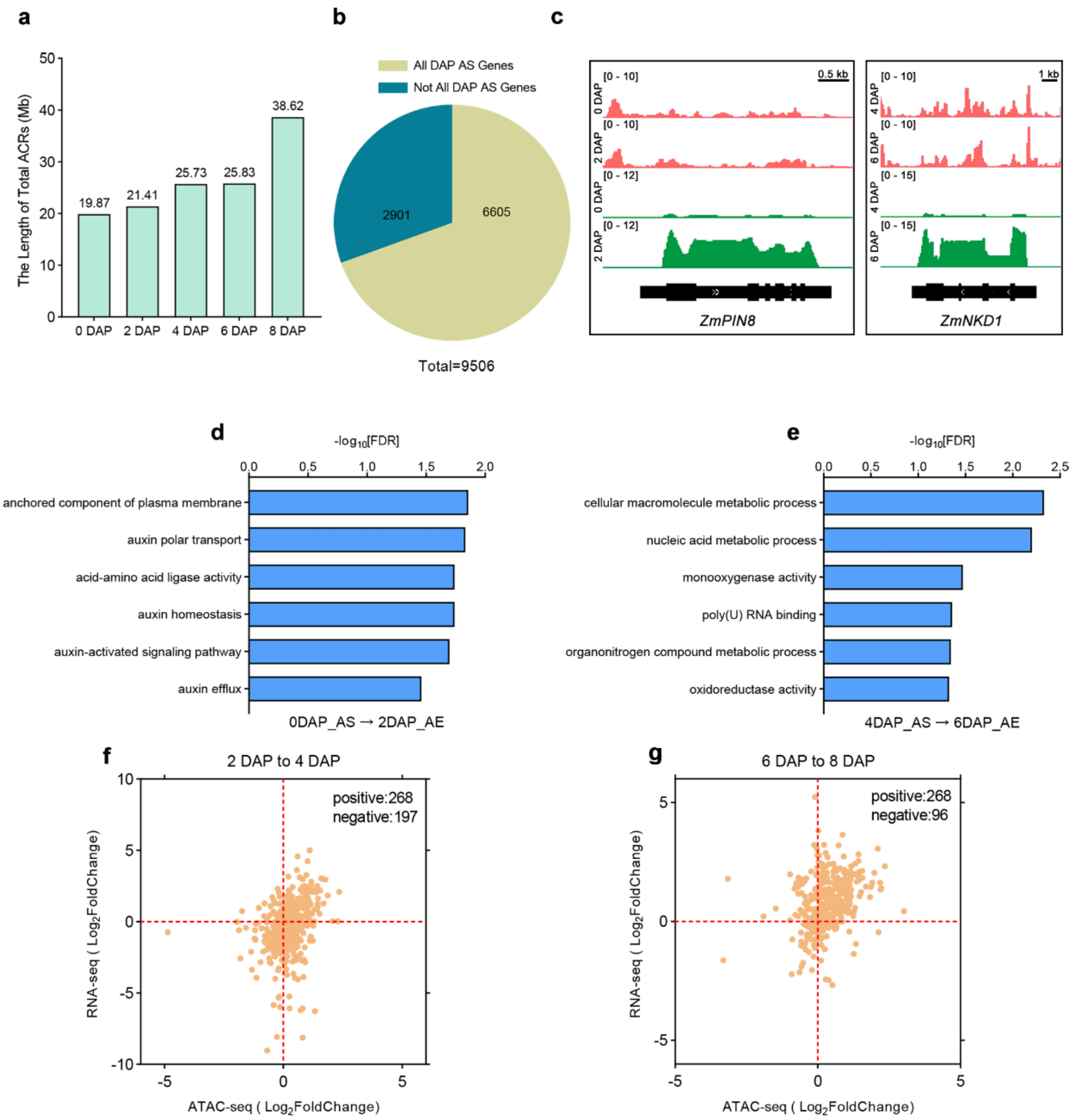

Figure S5. Chromatin accessibility and gene expression changes in seed development. **(a)** Bar chart show the length of total ACRs. **(b)** The pie chart of AS and AE distribution. **(c)** The ZmPIN8 and ZmNKD1 from AS to AE. Red: ATAC-seq signal, green: RNA signal. **(d)** and **(e)** Gene Ontology analysis of AS/AE change genes. **(f)** and **(g)** Correlation between changes in ACRs and gene expression for shift genes.

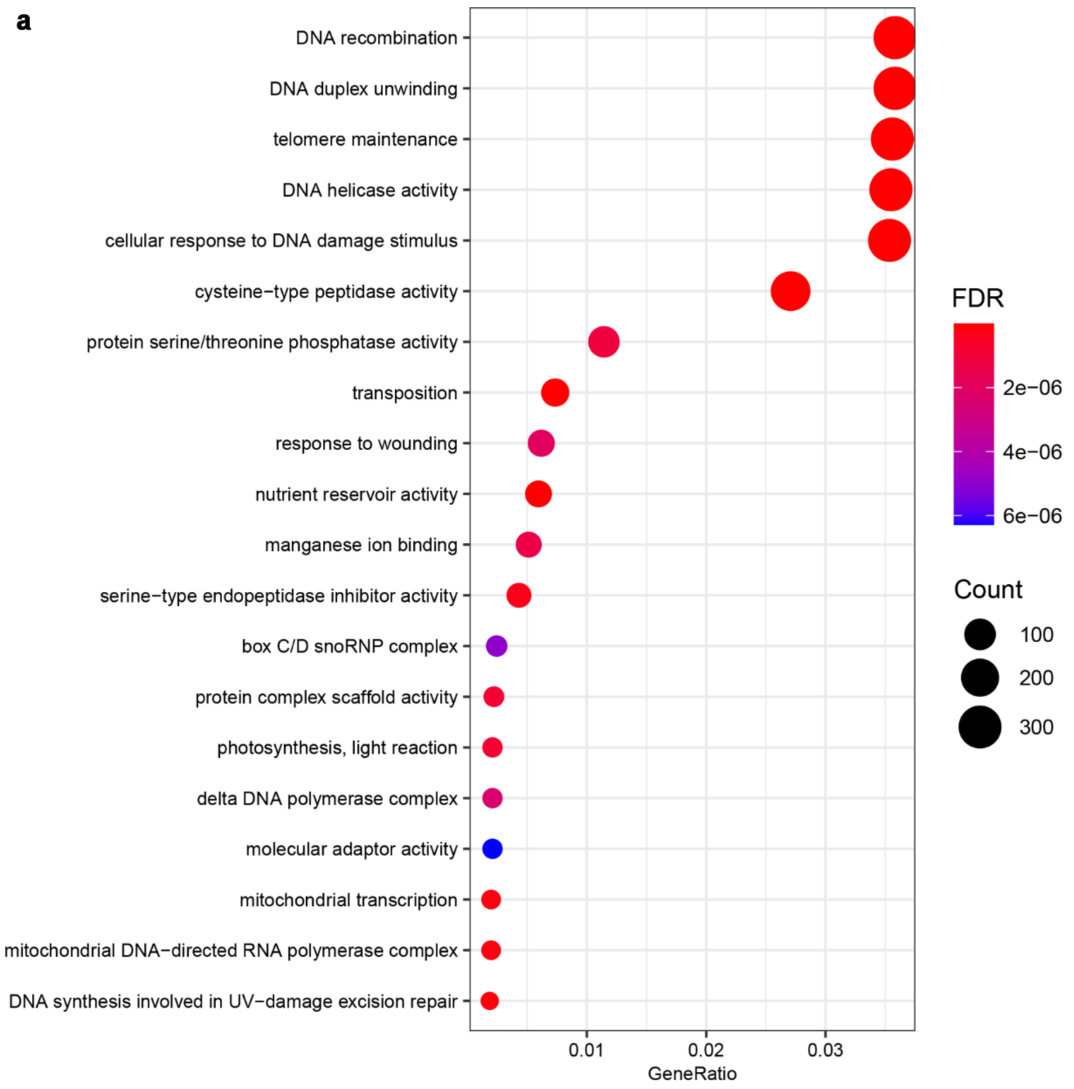

Figure S6. Gene ontology analysis of silenced and inaccessible genes. (a) Bubble plot of GO enrichment results for inaccessible and silenced genes during 0 DAP to 8 DAP (FDR < 0.05).

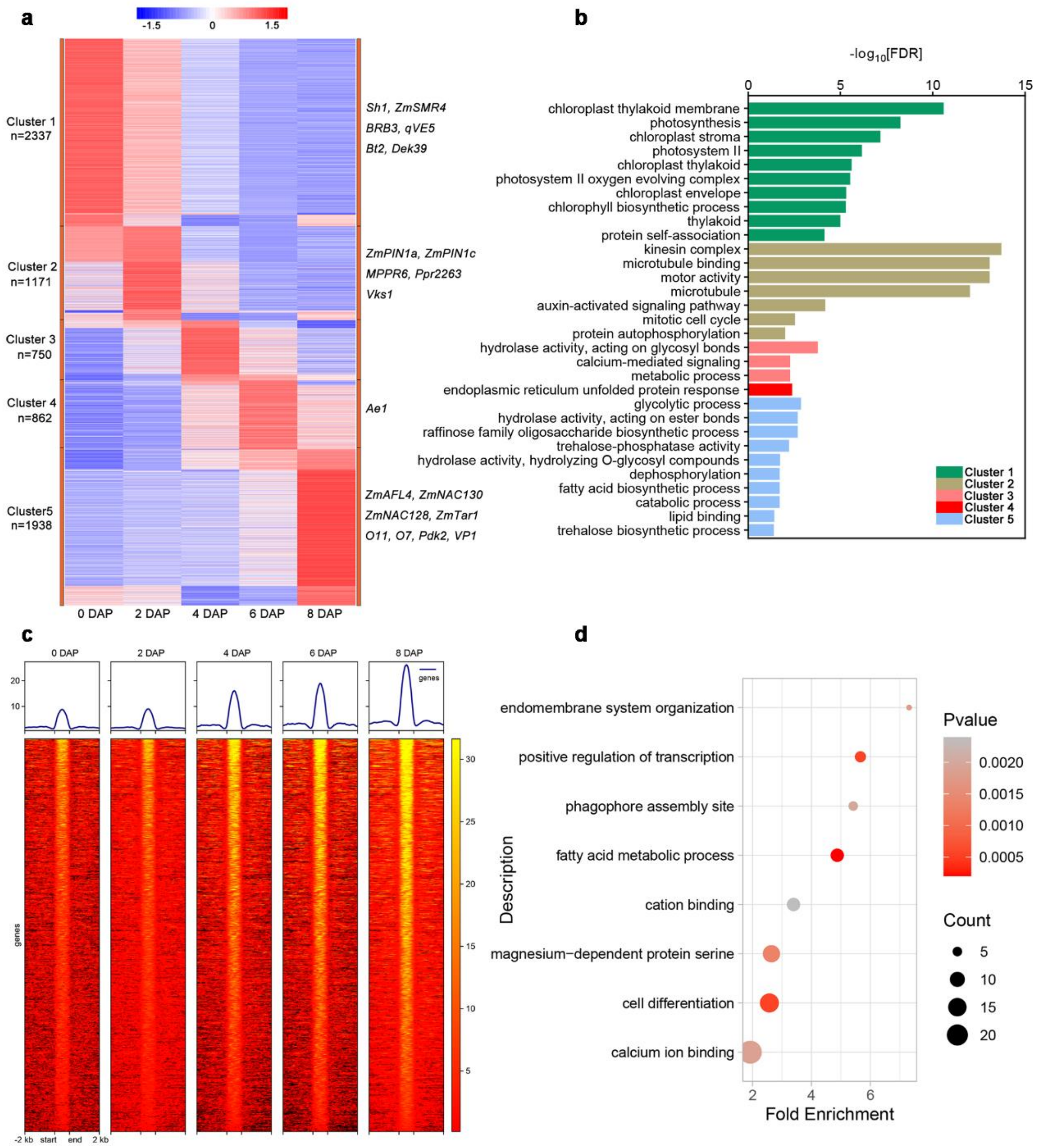

Figure S7. Stage specific gene expression from 0 DAP to 8 DAP. (a) Heatmap of relatively specific expressed genes (containing significant pACR accessibility) in different periods. **(b)** Gene Ontology (GO) analysis of different clusters (FDR < 0.05). **(c)** Increased ACRs from 0DAP to 8DAP. **(d)** Gene Ontology (GO) pathways involved in enhanced ACRs.

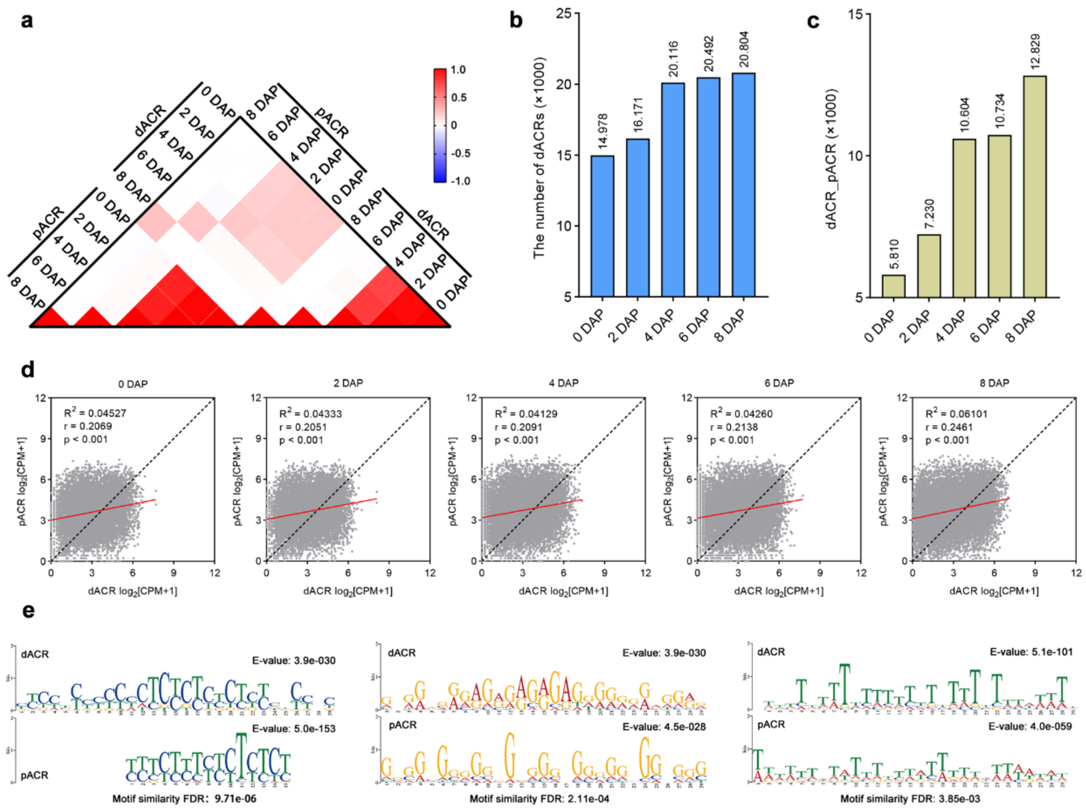

Figure S8. The dACR of development seed. (a) Heatmap of Spearman correlation between chromatin accessibility of pACR and dACR. **(b)** Bar plot of dACR number statistics at 0, 2, 4, 6, 8 DAP. **(c)** Bar plot of the number statistics with both dACR and pACR genes. **(d)** dACR and pACR signal correlation plot of 0, 2, 4, 6, 8 DAP. *r*: correlation coefficient, R^2: coefficient of determination. **(e)** Motif similar between pACR and dACR.

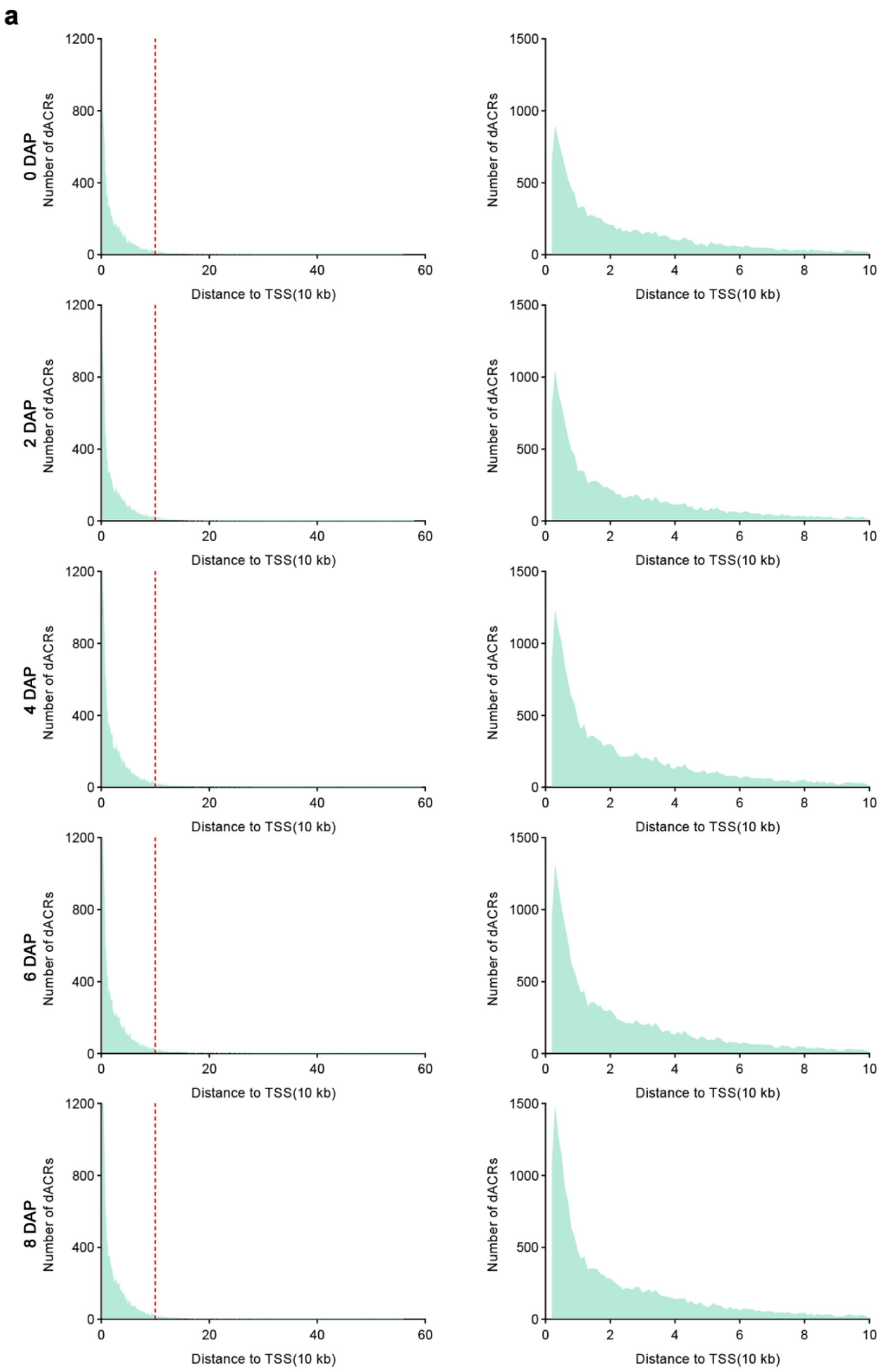

Figure S9. The distribution of dACR in the early stages of maize seed development. (a) The distance of dACR to the nearest TSS. The red line is located at 10.

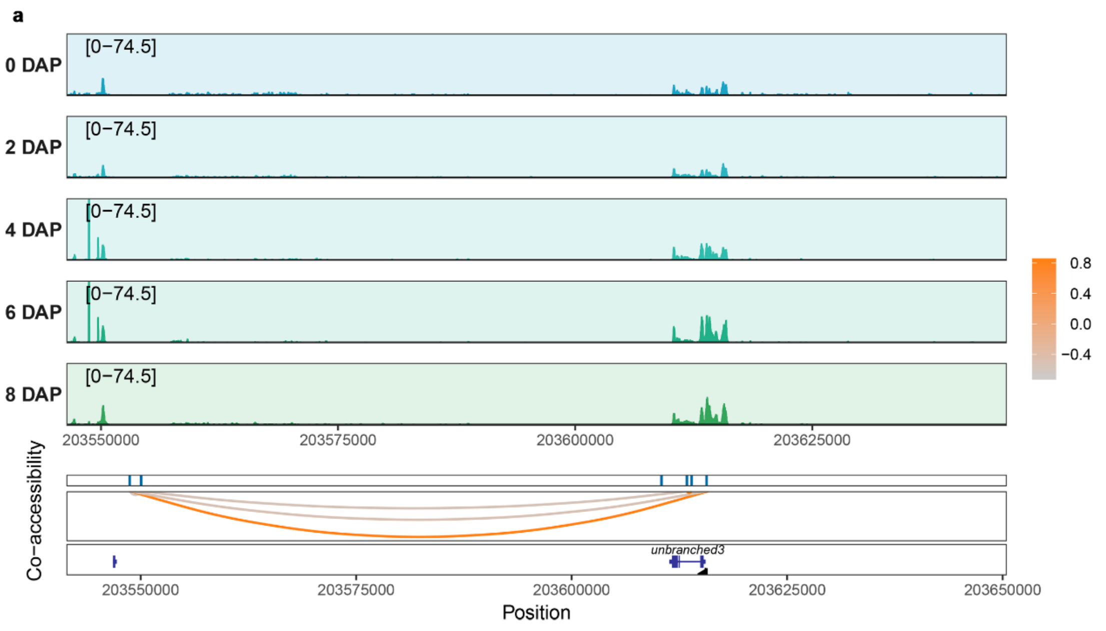

Figure S10. Co-accessible analysis of *UB3*. (a) Predicted co-accessible plot of *Unbranched3* across 0 DAP to 8 DAP chromatin accessibility dataset.

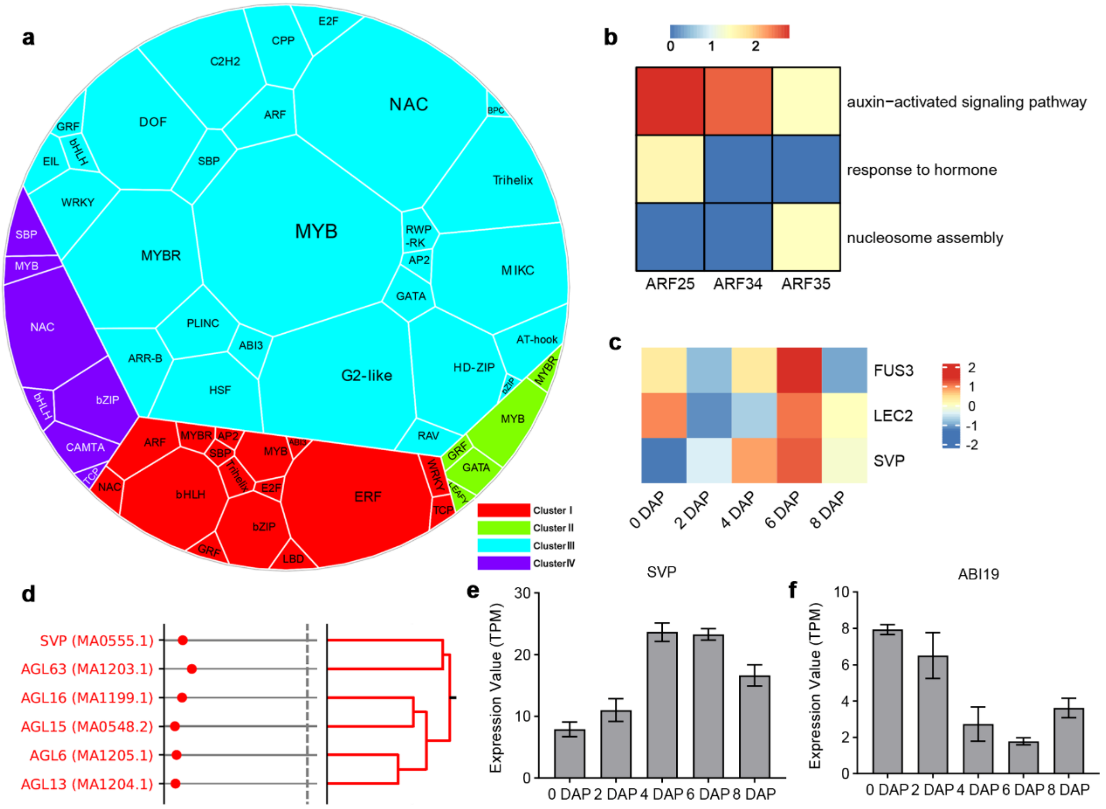

Figure S11. Distribution of transcription factor footprints. (a) Distribution of dominant transcription factors in the four clusters. **(b)** The GO analysis of ARF25, ARF34, ARF35 binding genes, from footprints analysis. **(c)** Heatmap of binding scores for central transcription factor footprinting. **(d)** Analysis of similarity among AGL transcription factor binding motifs. **(e)** and **(f)**

FPKM values of SVP and ABI19 from 0 DAP to 8 DAP.

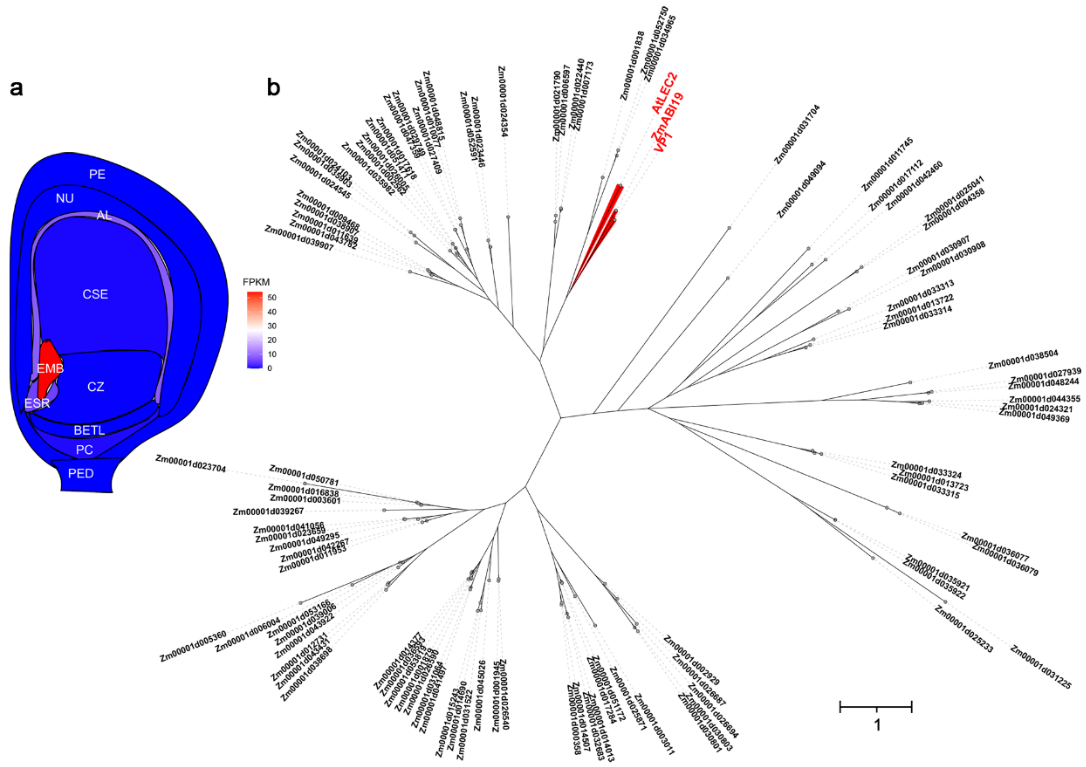

Figure S12. LEC2 homology analysis. (a) The ZmABI19 expression heatmap in maize seed at 8 DAP. **(b)** The maximum likelihood phylogenetic tree was constructed using full-length B3-domain transcription factors from maize and LEC2 transcription factors from Arabidopsis.

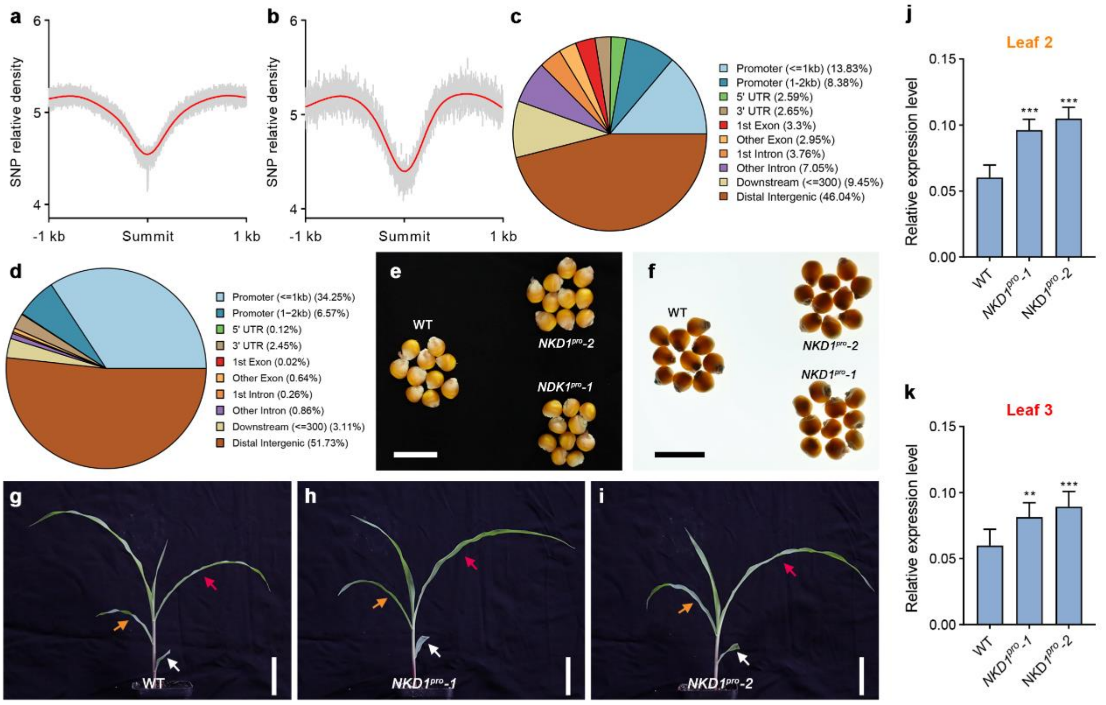

Figure S13. Variations in chromatin accessible regions. **(a)** Relative enrichment of single nucleotide polymorphisms (SNPs) for 1 kb regions flanking binding sites of transcription factors. **(b)** Relative enrichment of single nucleotide polymorphisms (SNPs) for 1-kb regions flanking ACR summits. **(c)** Distribution of trait-associated SNPs in maize seed ACRs. **(d)** Distribution of transposons in maize seed ACRs. **(e)** and **(f)** The mature seed phenotypes of WT, *NKD1^{pro}-1*, and *NKD1^{pro}-2*. Scale bar = 2 cm. **(g)** – **(i)** Fourteen-day seedling phenotypes of WT, *NKD1^{pro}-1*, and *NKD1^{pro}-2*. The white arrows indicate leaf 1, the orange arrows indicate leaf 2, and the red arrows indicate leaf 3. Scale bar = 5 cm. **(j)** and **(k)** The relative expression of *NKD1* in leaf 2 and leaf 3 between WT and *NKD1^{pro}*.
