# Supplementary material for: The chromatin accessibility landscape during early maize seed development
Source: Plant J. 2025 Mar 24;121(6):e70073. doi: 10.1111/tpj.70073 (PMC11932762; doi:10.1111/tpj.70073)
Supplement: Supplementary file 2 — Table S1. Summary of data generated in this study. [file TPJ-121-0-s001.docx]

**Table S1 Summary statistics of ATAC-seq and RNA-seq**

| No | Sample | Type | Length (nt) | Total reads | Aligned reads | % |
| --- | --- | --- | --- | --- | --- | --- |
| 1 | 0 DAP rep1 | ATAC-seq | 150 | 37324649 | 35,820,465 | 95.97 |
| 2 | 0 DAP rep2 | ATAC-seq | 150 | 40262283 | 39,118,834 | 97.16 |
| 3 | 2 DAP rep1 | ATAC-seq | 150 | 36891053 | 36,267,594 | 98.31 |
| 4 | 2 DAP rep2 | ATAC-seq | 150 | 33788250 | 33,480,776 | 99.09 |
| 5 | 4 DAP rep1 | ATAC-seq | 150 | 44120959 | 43,512,089 | 98.62 |
| 6 | 4 DAP rep2 | ATAC-seq | 150 | 40435102 | 39,885,184 | 98.64 |
| 7 | 6 DAP rep1 | ATAC-seq | 150 | 42300074 | 41,800,933 | 98.82 |
| 8 | 6 DAP rep2 | ATAC-seq | 150 | 40359088 | 39,790,024 | 98.59 |
| 9 | 8 DAP rep1 | ATAC-seq | 150 | 29062705 | 28,702,327 | 98.76 |
| 10 | 8 DAP rep2 | ATAC-seq | 150 | 59674104 | 57,478,096 | 96.32 |
| 11 | 0 DAP rep1 | RNA-seq | 150 | 71132614 | 69418318 | 97.59 |
| 12 | 0 DAP rep2 | RNA-seq | 150 | 75323276 | 73741487 | 97.90 |
| 13 | 0 DAP rep3 | RNA-seq | 150 | 70922022 | 69290815 | 97.70 |
| 14 | 2 DAP rep1 | RNA-seq | 150 | 54093762 | 52698143 | 97.42 |
| 15 | 2 DAP rep2 | RNA-seq | 150 | 55821980 | 54303622 | 97.28 |
| 16 | 2 DAP rep3 | RNA-seq | 150 | 50257502 | 48890498 | 97.28 |
| 17 | 4 DAP rep1 | RNA-seq | 150 | 70278876 | 68367291 | 97.28 |
| 18 | 4 DAP rep2 | RNA-seq | 150 | 68971490 | 67405837 | 97.73 |
| 19 | 4 DAP rep3 | RNA-seq | 150 | 75085024 | 73440662 | 97.81 |
| 20 | 6 DAP rep1 | RNA-seq | 150 | 70773896 | 69223948 | 97.81 |
| 21 | 6 DAP rep2 | RNA-seq | 150 | 71952250 | 70239786 | 97.62 |
| 22 | 6 DAP rep3 | RNA-seq | 150 | 72223762 | 70410946 | 97.49 |
| 23 | 8 DAP rep1 | RNA-seq | 150 | 69953312 | 68295419 | 97.63 |
| 24 | 8 DAP rep2 | RNA-seq | 150 | 58638782 | 57178676 | 97.51 |
| 25 | 8 DAP rep3 | RNA-seq | 150 | 72312506 | 70548081 | 97.56 |

Note: DAP: days after pollination
